# Forward and backward walking share the same motor modules and locomotor adaptation strategies

**DOI:** 10.1016/j.heliyon.2021.e07864

**Published:** 2021-08-23

**Authors:** Magdalena Zych, Annalisa Cannariato, Paolo Bonato, Giacomo Severini

**Affiliations:** a School of Electrical and Electronic Engineering, University College Dublin, Belfield, Dublin, Ireland; b Department of Physical Medicine & Rehabilitation, Harvard Medical School, Spaulding Rehabilitation Hospital, 300 First Ave, Charlestown, Boston, MA, 02129, USA; c Wyss Institute for Biologically Inspired Engineering, Harvard University, Boston, MA, USA; d Centre for Biomedical Engineering, University College Dublin, Dublin, Ireland

**Keywords:** Locomotor adaptations, Split-belt treadmill, Muscle synergies, Neuromechanical modeling

## Abstract

Forward and backward walking are remarkably similar motor behaviors to the extent that backward walking has been described as a time-reversed version of forward walking. However, because they display different muscle activity patterns, it has been questioned if forward and backward walking share common control strategies. To investigate this point, we used a split-belt treadmill experimental paradigm designed to elicit healthy individuals’ motor adaptation by changing the speed of one of the treadmill belts, while keeping the speed of the other belt constant. We applied this experimental paradigm to both forward and backward walking. We analyzed several adaptation parameters including step symmetry, stability, and energy expenditure as well as the characteristics of the synergies of lower-limb muscles. We found that forward and backward walking share the same muscle synergy modules. We showed that these modules are marked by similar patterns of adaptation driven by stability and energy consumption minimization criteria, both relying on modulating the temporal activation of the muscle synergies. Our results provide evidence that forward and backward walking are governed by the same control and adaptation mechanisms.

## 1 Introduction

Gait is a stereotyped motor behavior that presents a high level of similarity among different individuals. Likely contributing to this similarity is the fact that the fundamental modules underlying the control of gait appear to be present since birth [1] and to combine dynamically during development [2]. The biomechanical stereotypes of walking are consistent between walking directions, as we observe remarkable similarities between forward walking (FW), that is one of the most common activities of daily living, and backward walking (BW), that is a seldom utilized behavior. BW is similar to a simple time-reversal of FW, both visually [3] and kinematically as well as kinetically [4, 5], to the point of raising the question of whether shared, if not identical, neural and spinal circuitry controlling both FW and BW are present in humans and are just temporally inverted to produce the two motor behaviors [6]. However, electromyographic studies have shown that muscle activations during FW and BW are markedly dissimilar [7, 8] and that BW muscular activations are not simple time-reversals of the FW ones.

Recent studies on muscle synergies have suggested that a two-level organization (at the supra-spinal and spinal level) underlies the control of locomotion [9, 10], where spinal modules are recruited differently depending on the task and the task parameters. These spinal modules appear to be well represented by the muscle synergy module components of the non-negative matrix factorization-based muscle synergy analysis [11, 12, 13]. Although direct physiological evidence of the existence of these spinal circuits cannot currently be derived in humans, recent studies have reinforced the hypothesis behind their existence by showing that the spatial muscle synergy modules obtained by decomposing neuromuscular activity are shared across different tasks [14, 15] and are stable during the response to perturbations [16, 17]. In light of this evidence, it is possible that FW and BW share, at least partially, the same muscle synergy modules, given the similar role that some muscle groups perform during the two tasks [18]. However, studies employing dimensionality reduction techniques have so far shown marked differences in the organization of the spatial patterns of activation of the lower limb muscles in FW and BW while finding consistent temporal patterns [8, 19, 20].

A methodology that is often utilized to investigate the neural and spinal circuits involved in the control of locomotion and lower limb muscles is motor adaptation. The study of locomotor adaptation in FW and BW has, so far, delivered conflicting results. It has been shown that adaptations to belts running at different speeds during split-belt treadmill walking do not transfer between the forward and backward directions [21] suggesting a complete independency between the circuits controlling these two behaviors. On the other hand, podokinetic after-rotations, which are residual curved trajectories that appear during walking after periods of training on a rotating platform, do transfer between directions [22], indicating some level of shared control. Several processes, such as balance maintenance, natural bias toward symmetry and metabolic cost minimization contribute to the formation of adaptation motor plans [23] and their interplay is likely to be a key factor in enabling the transfer of the adaptation observed for a given walking task to a different walking condition. However, it is not clear if the same principles and hierarchy among these adaptation processes are shared between FW and BW.

In this work, we shed light on the neural control of forward and backward locomotion by relying on a split-belt locomotor adaptation experimental paradigm to investigate similarities and dissimilarities between FW and BW control. Our goal was to determine whether the same processes, and possibly the same neural pathways, contribute to the generation of both behaviors. Hence, we asked a group of healthy individuals to perform two split-belt treadmill adaptation experiments, one walking forward and one backward, in two different days in a randomized order. In both these experiments, participants initially walked for 5 min with the belts running at the same speed (tied-belt, baseline phase), then walked 10 min with the right belt running at twice the speed of the left belt (split-belt, adaptation phase) and concluded each experiment by walking for 5 min again with the belts tied (post-adaptation phase). During the experiments, we recorded the kinematics and kinetics of the lower limbs together with the electromyographic (EMG) activity of eight muscles on each leg.

We used an EMG-driven neuromuscular model [24] to estimate the activity of 33 unilateral Musculo-Tendon Units (MTUs) during the different phases of the two experiments, starting from the EMG recordings of 8 muscles per leg. Of these 33 unilateral MTUs, 26 were mapped from the EMG recordings, and 7 completely synthesized. The activity of the 26 mapped MTUs was used to calculate the muscle activation synergies of the two tasks and their evolution during the different phases of the two experiments. We used the modeled activations rather than the actual EMGs to increase the neuromuscular resolution of our analysis and to better estimate the level of activation of the different muscles by tying them to the kinematics and kinetics of the task, thus reducing the variability associated with EMG recordings. Moreover, this modeling approach allowed us to use the activations of all the MTUs for estimating the changes in energy consumption during the different phases of the two experiments using a model of energy consumption that has been shown to provide energy consumption estimates that are in accordance with experimental data [25, 26].

We found that FW and BW are obtained via the activation of identical sets of MTU synergies encompassing the flexor and extensor muscles of the hip, knee and ankle. Some of the synergies appear to share the same role in both tasks, and all of them are maintained in their composition during both FW and BW adaptation processes. Both adaptation processes were controlled via high-level strategies aiming at preserving stability and minimizing energy consumption. Our results show that FW and BW share remarkable similarities at the neurophysiological level, both in terms of the high-level strategies utilized for modulating both behaviors and of the low level muscular coactivation modules, that appear to be equivalent for the two walking directions.

## 2 Methods

### 2.1 Participants

Six healthy individuals (4 females, height 1.64 ± 0.02 m; body mass 61.4 ± 3.9 kg, age 26.5 ± 3.3 years) participated in this study. Volunteers had no orthopedic, neurological, or cognitive impairments. All procedures were conducted in conformance with the Declaration of Helsinki. The study protocol was approved by the Ethical Committee of Harvard University. All participants provided informed consent before participating in the experiments. All the data collections were performed at the Wyss Institute for Biologically Inspired Engineering in Boston.

### 2.2 Experimental protocol

The experimental protocol consisted of two sessions each including one experiment on either FW or BW on a split belt-treadmill (Bertec, Ohio, US). The sessions were held on different days and the order of the sessions was randomized. During the different experimental procedures, the belts could perform in two ways: “tied”, when the belts were set to run at the same speed, or “split” when the belts were set to run at different speeds. Before the beginning of each experimental session a static trial was collected for model scaling purposes. Each experiment (FW and BW) consisted of three phases: baseline, adaptation, and post-adaptation. During the baseline phase the participants walked for 5 min with the belts tied and set at a speed equal to 0.4 m/s. This was followed by 10 min of adaptation phase, where the belts were split, with the right side running twice as fast with respect to the left one, at a speed of 0.8 m/s. The last phase of each experiment consisted of a 5-minutes post-adaptation performed with the belt tied at a speed of 0.4 m/s. Between each phase of each experiment the belts were stopped and, after approximately one minute, restarted to begin the new session. During all procedures, participants wore a harness as a security measure. The harness did not provide weight support. Participants were asked not to hold on the handrails during the trials and were encouraged to look in front of them during each, tied or split belt, walking bout.

### 2.3 Data acquisition

Kinematic data were collected with the Vicon system (Denver, US) using the standard lower limb “plug-in gait” setup [86] and sampled at 120 frames per second. The split-belt treadmill was instrumented with force plates under both belts to record kinetic data, sampled at 1200 Hz. The activity of the muscles was recorded using a 1200 Hz sampling frequency using a Delsys (Boston, US) Trigno system. Sixteen muscles (8 muscles bilaterally) were recorded: Tensor Fasciae Latae (TFL), Rectus Femoris (RF), Vastus Lateralis (VL), Tibialis Anterior (TA), Gluteus Maximus (Gmax 2), Biceps Femoris Long (BFl), Gastrocnemius Lateralis (GL), Soleus (Sol). The placement of the EMG sensors was performed according to SENIAM recommendations [87]. The muscles were selected for their role in flexion/extension of the hip, knee and ankle, although the TFL also contributes to hip abduction. This setup is consistent with another study on robot-induced locomotor adaptation [17].

### 2.4 Data pre-processing

All biomechanical modeling performed in this work is based on the OpenSim software [88]. The data was prepared for OpenSim using the MOtoNMS toolbox [89]. Within the toolbox, the EMG signals were digitally filtered with a 2^nd^ order Butterworth band-pass filter with a cut-off frequency of 30–300 Hz and were then rectified. Envelopes were then obtained by applying a 2^nd^ order Butterworth low-pass filter with cut off frequency of 6 Hz. The envelopes were normalized to the maximum value of each muscle for each session. For consistency with the EMG envelope extraction, a 6 Hz low-pass filter was applied to the kinematic and kinetic data. After pre-processing, the first, middle, and last 10 gait cycles from each of the phases (baseline, adaptation, post-adaptation) were extracted for further analysis. Gait cycles were segmented using the initial contact of the left leg. Initial contact was determined as the instant when the vertical force reached above 20 N [90]. Using the Opensim software [88], a modified generic neuromuscular model (gait2392) with 23 degrees of freedom (DoF) and 66 musculo-tendon units (MTUs) was scaled to the anthropometric measures of each subject, using the static trials recorded before each session. Joint angles and moments were calculated from the scaled models by applying the inverse kinematics and inverse dynamics routines present in Opensim to each of the extracted walking bouts. The muscle analysis tool [88] was used to calculate the contribution of each muscle into the torques calculated for each degree of freedom. These data are needed as input for the CEINMS software.

### 2.5 Step symmetry index and step timing

Adaptation to split-belt treadmill walking is typically assessed by analyzing the changes in Step length symmetry through the course of the different phases of the experiment [23, 36, 91]. To confirm the presence of the typical step length adaptation processes in our experiments, the step length symmetry was calculated as:

(1) $$symmetry=\frac{sR-SL}{sR+sL},$$

where *sR* and *sL* are the Step length of the right and left foot accordingly [21]. Values smaller than 0 indicate that the step length of the right, fast leg is shorter than the left, slow, one, and vice-versa. Step length was calculated as the difference between the longitudinal position of the ankle markers at the heel strike. We assessed changes in step timing during and after adaptations by extracting the instant of initial contact for the fast (right) limb within the time-normalized gait cycle of the slow limb. For this metric, a value of 50 indicates the natural timing during unperturbed walking.

### 2.6 Kinematics and kinetics

The kinematics and kinetics patterns were time normalized to the length of each gait cycle. To evaluate the changes in joint angles and moments due to split-belt adaptation and de-adaptation with respect to normal, tied-belt, walking we calculated the R^2^ measure between the average late baseline (last 10 steps of the baseline phase) angle and moment patterns with those of each of the steps of the 10-steps portions extracted from the three phases of both experiments.

### 2.7 Stability

In our analyses, we evaluated changes in longitudinal gait stability during the adaptation to both split-belt scenarios. For this analysis we used as metric the margin of stability (MoS) at foot off (FO). The MoS parameter was calculated as the difference between the base of support (BoS) and the position of the extrapolated Centre of Mass (XCOM) [42]. FO was defined, in both experiments, as the moment when the ground reaction force applied by a foot dropped below 20 N [90]. The BoS at FO was calculated, for FW, as the distance between the position of the toe marker in the leading and the trailing foot. For BW walking, the BoS was calculated as the difference between the position of the calcaneus marker in both the leading and trailing feet. The XCOM parameter accounts for changes in the velocity of the projected COM in the walking direction and is calculated as follows:

(2) XCOM=COM+VCOMgl,

where *l* is the length of the leg, *g* is the gravity acceleration and *V*_*COM*_ is the velocity of the COM [42]. In our calculations, the COM position and velocity were obtained using the BodyKinematics tool in OpenSim. To examine how the position of the XCOM shifted and, consequently, the MoS changed with respect to the changing BoS during both adaptation phases, we introduced the measure of the ratio between the MoS to BoS (r = MoS/BoS). The MoS and ratio parameters were calculated for each Step of the extracted phases.

### 2.8 EMG-informed musculoskeletal modelling

In our analysis we used the EMG-informed neuromuscular model developed in the CEINMS toolbox [24] to estimate the neural excitations relative to all the 66 MTUs included in the biomechanical model, starting from the kinematics, kinetics and the limited set of EMG channels that were recorded during the data collections. The CEINMS toolbox uses an activation dynamic model to extract the neural activations from the muscle excitations and calculates the contraction dynamics using a modified Hill-type muscle model to estimate the muscle forces. At the beginning of this process, a calibration procedure was used in order to find the subject-specific values for the parameters describing the different MTUs within the activation and musculotendon contraction dynamics models, as in [48, 92]. The calibration optimization was used to adjust the non-linear MTU parameters by minimizing the difference between the predicted and measured joint moments. The parameters describing the activation dynamics were adjusted globally for all MTUs. The neural activation coefficients were restricted between -1 and 1 and the shape factor describing the neural to muscle activation was restricted between -3 and 0.

In the model the MTUs were divided into 10 functional muscle groups. The strength coefficient, which is a factor that multiplies the maximal isometric force of each muscle, was adjusted within 0.5–3 for each muscle group. The MTUs were either mapped from the recorded EMGs or synthesized if there was no EMG recorded for them. Fifty-two musculotendon units (MTU) were mapped, with different weights (Table S2), from the recorded EMGs according to their functional and innervation properties. Sixteen MTUs were mapped directly from the recorded EMGs (see *Data Acquisition*), while the other 36 (18 per side) included: Gluteus Medius (Gmed 1,2 & 3), Gluteus Minimus (Gmin 1, 2 & 3), Gluteus Maximus (Gmax 1 & 3), Biceps Femoris Short (BFs), Semimembranosus (Semimem), Semitendinosus (Semiten), Sartorus (Sar), Peroneus Brevis (Perbrev), Longus (Perlong), Tertius (PerTert), Gastroecnamius Medial, Vastus Medialis (VM), and Intermedialis (VI). An additional 14 MTUs (7 per side) were synthesized through the optimization process present in CEINMS: Psoas, Iliacus, Adductor Brevis, Longus, Magnus (3 MTUs). The optimal fibre length at the maximum activation and the tendon slack length were optimized to in a ±15% range around the initial values of the scaled model for each MTU.

The calibration was performed using the data extracted from the middle 10 steps of baseline. The optimization procedure was solved for the hip, knee, and ankle angles on the sagittal plane. After the calibration procedure, the calibrated subject-specific models were used to simulate the neural activation patterns calculated from the activation dynamics and adjusted EMGs for all the remaining walking bouts extracted in the three phases of both experiments.

For this estimation we utilized a hybrid algorithm, available in CEINMS, combining the static optimization algorithm and the EMG-forward dynamic modeling [93]. The algorithm adjusts the recorded EMG signals while minimizing the difference between experimental and predicted joint moments. This procedure is based on the optimization of the following objective function:

(3) $$F_{obj}=\alpha F_{trackMoM}+\beta E_{sumEXC}+\gamma E_{trackEMG}$$

where α = 1, β = 2, and γ = 10 are weighting coefficients, F_trackMoM_ is the total error between the estimated and experimental joint moments, E_trackEMG_ is the error for the adjusted muscle excitations estimated through the EMG-driven algorithm and E_sumEXC_ is the sum of the squared estimated excitations predicted with Static Optimization formulation. Weighting coefficients have been chosen empirically as the values that yielded the best fit values across subjects.

### 2.9 Metabolic cost

In our analysis we characterized the changes in the energetic cost associated with the adaptation processes. In order to do so we estimated the metabolic cost during the different phases of both experiments from all the 66 MTUs using the model developed by Umberger et al. [25] and by applying the slow-twitch ratio model by Bhargava et al [26]. In this model, the energy expenditure of a single MTU is defined as the sum of the activation heat rate, the maintenance heat rate, the length change heat rate, and the mechanical work rate of the contractile element. All these parameters are calculated from the simulated activations, the adjusted EMGs, and the mechanical properties of the different MTUs. The gross metabolic rate for a single gait cycle was calculated as the sum of the integrated total energy from all the MTUs. The energy pattern for each cycle was time normalized before the integration to obtain metabolic power. The metabolic power was calculated separately for the MTUs of each leg.

Furthermore, we calculated the side-specific metabolic rate during different gait phases of the fast leg, namely early and late stance (Est, Lst) and early and late swing (Esw, Lsw). For this analysis, the beginning and end of the stance phase were identified as the instants of initial contact and foot-off, estimated as the instants when the ground reaction force raised above 20 N and fell again below 20 N respectively [90]. The time points dividing the stance and swing phases into its early and late stages were selected as the mid-time of the whole stance and swing phases.

### 2.10 MTU activation synergies

We extracted, in both experiments, the muscle synergy modules and activation patterns relative to the MTUs, similarly to what usually done in muscle synergies analysis [17]. In this analysis we only used the MTUs that were mapped from the experimental data and we excluded those which were synthesized to avoid the appearance of synergies that were fully synthetic and not based, at least partially, on recorded data. The synergies were extracted from the MTU activations estimated for each sub-phase of each experiment using the semi-fixed model that we developed in a previous work [27]. In this model, a set of reference weights *W*^*ref*^ are extracted, for each experiment, from the MTU activations relative to the late baseline phase, using the standard non-negative matrix factorization algorithm [94]. These reference weights are used to determine the range over which the contribution of each MTU to each synergy is allowed to vary by the semi-fixed algorithm when estimating the synergies for all the other phases of both experiments. Specifically, given:

(4) $$MTU_{m,s}^{Ref}\approx \;W_{m,n}^{Ref}\cdot H_{n,s}^{Ref}$$

where $W_{m,n}^{Ref}$ and $H_{n,s}^{Ref}$ are respectively the synergy weights and activation patterns extracted by applying the NMF algorithm on the matrix $MTU_{m,s}^{Ref}$ represented in this case by the MTUs estimated during the lb portions of the experiments, with the matrices $W_{m,n}^{Ref}$ and $H_{n,s}^{Ref}$ scaled so that $0<\;W_{m,n}^{Ref}<1$, the semi-fixed synergies model estimates the MTU synergies for each phase of both experiments as:

(5) $$MTU_{m,s}^{Exp}\approx \;W_{m,n}^{Exp}\cdot H_{n,s}^{Exp}$$

By applying the standard multiplicative update rule of the NMF while bounding the variability of the single components of the weight matrix $W_{m,n}^{Exp}$ by a tolerance parameter δ so that:

(6) $$\max\left(0;W_{m.n}^{Ref}-\delta \right)<W_{m,n}^{Exp}<\text{min}\left(W_{m.n}^{Ref}+\delta ;1\right)$$

While the parameter $H_{n,s}^{Exp}$ is completely left free to change and capture the variability of the changes in MTU activity in the different phases of the experiments. The tolerance parameter δ was set to 0.15 in our analysis, indicating that the single weights of each MTU were allowed a maximal variability of 15% around its value estimated during baseline. The choice of the semi-fixed algorithm is based on the numerous observations by us and others [17, 27, 29, 31, 68] indicating that upper and lower limb motor adaptations are well represented by modulation of the activation patterns of fixed or barely changing synergies, while changes in weights (or recruitment of additional/different synergies) are required only when biomechanical task demands change [68]. The synergy extraction was performed unilaterally, separating the MTUs on the left and right leg. We extracted 5 unilateral modules [64], from each of the 10-steps walking bouts extracted from the three phases of the two experiments. The number of modules was kept fixed across individuals and conditions. The quality of the reconstruction was estimated for each phase of each experiment using the R^2^ parameter calculated between the original and reconstructed MTU activations. We evaluated how the activation patterns of the MTU synergies changed during the different phases of each experiment using different parameters. First, we calculated the reference step as the average $H_{n,s}^{Ref}$ across the 10 steps of *lb* of each experiment. Then we segmented and time-normalized each step in the $H_{n,s}^{Exp}$ activation matrix. We estimated the overall changes in the similarity between the average $H_{n,s}^{Ref}$and the activation patterns of each step using Pearson's coefficient. We then evaluated how those overall changes in similarity reflected in changes in timing and magnitude of activation. The timing was evaluated by calculating the delay (calculated as the point of maximal cross-correlation) between the reference baseline activation and the activation of each step. Changes in overall amplitude were calculated as the percentage changes between the integrated activation of the reference step and the integrated activation of each step.

### 2.11 Model evaluation

The evaluation of our modeling approach was based on the evaluation practices suggested for OpenSim models. The assessment of the quality of the model's inverse kinematics, for each of the subjects for each session, was based on the root mean square (RMS) function that is built in Opensim that calculates the RMS between the experimental marker positions and virtual positions extrapolated from the calculated kinematics. For FW the average RMS was kept under 1 cm and the maximum marker error was less than 2 cm, for BW these values were 2 and 4 cm respectively, which complies with OpenSim guidelines. To evaluate quality of the joint moments prediction performed by CEINMS, the R^2^ measure between the joint moments obtained by applying the inverse dynamics to the experimental data and the joint moments predicted by CEINMS was calculated. We found values of R^2^ exceeding 0.9 on average across subjects and experimental sub-phases.

## 3 Results

### 3.1 The same muscle synergies control FW and BW

We analyzed the composition of the muscle synergy modules during FW and BW using a model-based extension of the standard muscle synergies approach. We extracted the side-specific MTU synergies from 52 (26 per side) MTUs that were mapped from the experimental data using a EMG-informed neuromechanical model [24]. We estimated the activity of all the MTUs based on the kinematics and kinetics of each task and a subset of 16 (8 per side) EMG signals recorded during the experiments (see *Methods*).

We extracted 5 synergies from each side using both the standard non-negative matrix factorization algorithm (NMF) to analyze the synergies during unperturbed FW and BW at baseline, and a previously developed semi-fixed synergy algorithm [27] to analyze synergies during adaptation and post-adaptation. The rationale for using the semi-fixed algorithm is based on the multiple observations, by our group and others, that synergy weights do not change substantially during motor and locomotor adaptations [17, 27, 28, 29, 30, 31]. In the semi-fixed synergies model, the weights of the individual muscles in each synergy module are allowed to vary only to a limited extent around their baseline values (see *Methods)*.

**Figure 1:**
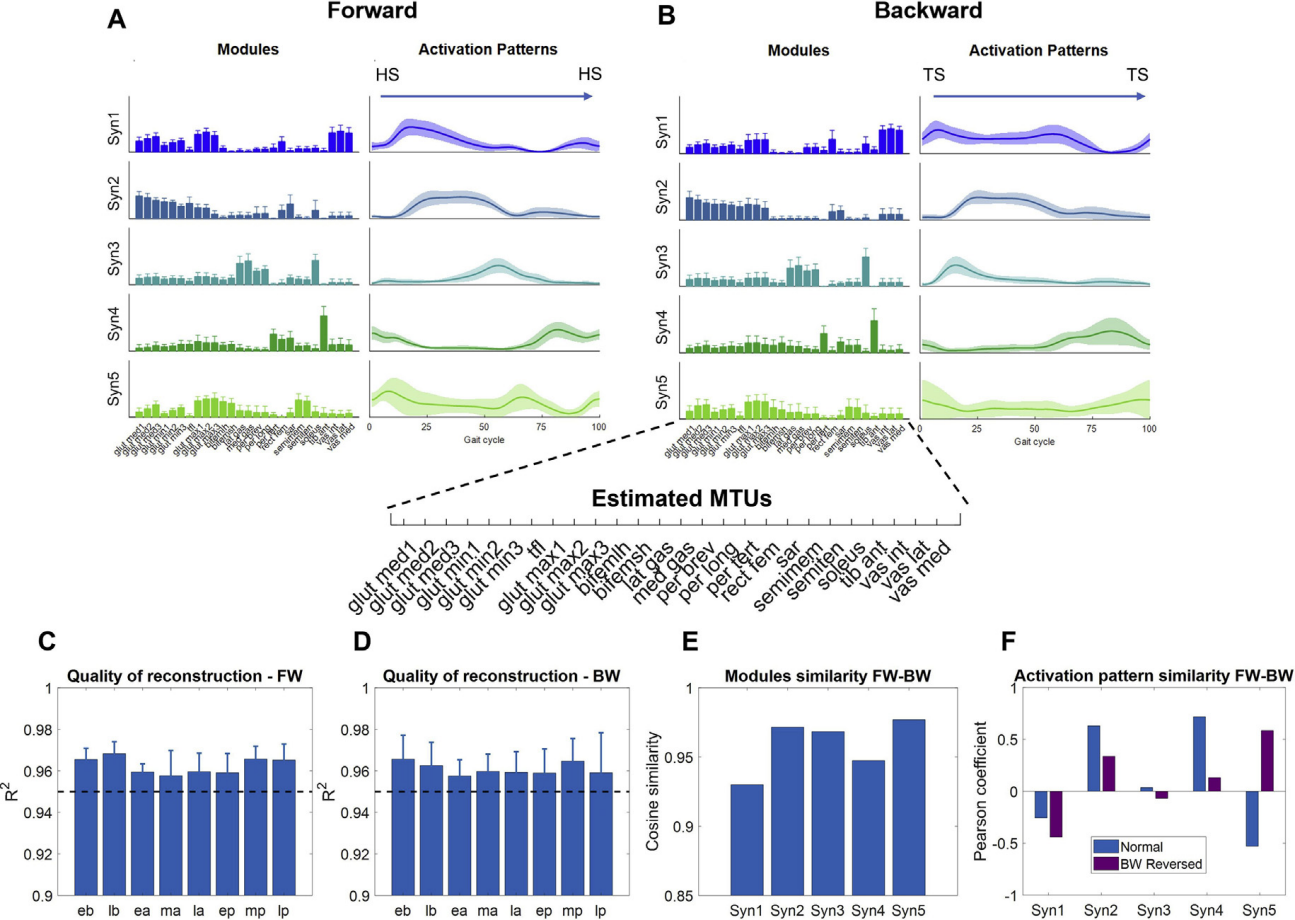
Synergy modules and activation patterns for FW and BW. The two top panels present the synergy modules and activation patterns extracted during the late baseline (last 10 steps) phase of FW (A) and BW (B). The modules and patterns are extracted from the activations of 26 MTUs mapped from the recorded EMG data. The modules are presented as bars showing the average and standard deviation values across subjects. For each subject, the representative modules were calculated as the average of the modules extracted from both legs. The activation patterns are plotted from heel-strike (HS) to heel-strike for FW and from toe-strike (TS) to toe-strike for BW. The solid line represents the average across subjects. The shaded area represents the standard deviation across subjects. Panels (C) and (D) show the quality of reconstruction for all phases of the FW and BW experiments respectively, obtained by extracting the synergies using the semi-fixed synergy algorithm. The quality of reconstruction is expressed using the R^2^ between the original activations and the reconstructed ones, for all the experimental phases (i.e., eb – early baseline, lb – late baseline, ea – early adaptation, ma – mid adaptation, la – late adaptation, ep – early post-adaptation, mp – mid post-adaptation, lp – late post-adaptation). In both plots the bars represent the average and standard deviation across subjects. The dashed black line represents a R^2^ = 0.95. Panel (E) shows the cosine similarity values between the average (across sides and subjects) modules extracted in late baseline for FW and BW. Panel (F) shows the similarity, calculated using the Pearson's correlation coefficient, between the average (across sides and subjects) activation patterns extracted during late baseline for FW and BW (blue bar) and for FW and the time-reversed activation pattern of BW (purple bar) segmented from heel-off to heel-off. This segmentation allows to compare FW stance and swing with the reverse of BW stance and swing.

We found (Figure 1) that 5 synergies were able to achieve good, by literature standards, EMG reconstruction quality with average (across subjects and muscles) R^2^ values >0.9 during all the phases of both experiments. Also, in both cases, the synergies extracted during FW and BW are almost identical, with cosine similarity for each synergy composition ranging between 0.93 and 0.98 for the baseline synergies. Furthermore, we found that 2 of the 5 synergies maintained similar activation patterns (Pearson Coefficient >0.5) during FW and BW (Figure S1).

The five synergies have specific composition and functional characteristics (from top to bottom in Figure 1), as identified in previous studies for both simulated and experimental data [32, 33, 34, 35], with prominent activity of the following muscles: Syn1) Quadriceps Femoris and Gluteus Maximum - this synergy is active to ensure hip stabilization during the loading response in FW and during all the stance phase in BW, where it is also the main contributor to backward propulsion; Syn2) Gluteus Medius, Gluteus Minimus and Tensor Fasciae Latae - this synergy follows roughly the same activation pattern for both FW and BW (Figure 1, Pearson Coefficient = 0.63 between the two conditions) and its main role is limb stabilization during mid stance; Syn3) Triceps Surae - this synergy characterizes the propulsive activity of the calf muscles during late stance and knee extension in FW while it controls the weight acceptance phase in BW walking; Syn4) Rectus Femoris, Tibialis Anterior and Peroneus, this synergy holds roughly the same role in FW and BW walking (Pearson Coefficient = 0.72 between the two conditions) by assisting early swing and controlling the foot landing preparation phase; Syn5) Gluteus Maximus and Hamstring, in FW this synergy is active twice, namely during initial contact and early stance to decelerate the leg and control the extension of the hip and during swing to stabilize the leading leg, while it is active during the whole gait cycle in BW, with a higher activation during late swing and initial contact, possibly as a contributor to limb stabilization and deceleration. We analyzed whether the MTU synergies activation patterns during baseline BW are similar to the time-reversed activation patterns during baseline FW, as observed in previous work for the kinematics of BW compared to FW [8]. We found that the BW synergy activations are dissimilar from the reversed FW activations (Figure 1 and S1) for Syn1-4, with values of Pearson Coefficient below 0.3, while they were similar for Syn5 (Pearson Coefficient = 0.52). It should be noted that the result on the similarity between FW and BW muscle synergy modules holds true for both the synergies estimated with the semi-fixed approach (Figure 1) and those estimated using the standard NMF, both on recorded EMGs (Figure S6A) and the reconstructed MTUs (Figure S6B), and that the synergies extracted from both the MTUs and the actual EMG recordings are remarkably similar across subjects and experiments (Figure S7).

### 3.2 Synergies adaptation

To assess whether FW and BW split-belt adaptations employ different adaptation strategies, we characterized adaptation in different domains, namely neuromuscular control, symmetry and stride timing, stride-to-stride stability and energy consumption. Considering the changes happening at the neuromuscular level during adaptation, the analysis presented in Figure 1 shows that split-belt adaptation is obtained without changing the composition of the muscle synergy modules. In our semi-fixed synergies analysis, we allow the single weights in the synergy modules to change by 15% around the values of the modules during baseline. The fact that we do not observe a drop in the quality of the reconstruction of MTU activity using this approach (Figure 1, panels C and D) suggests that the synergies modules obtained using the semi-fixed algorithm can well represent the muscular activity during adaptation and thus that the synergy modules do not change substantially during adaptation and post-adaptation.

**Figure 2:**
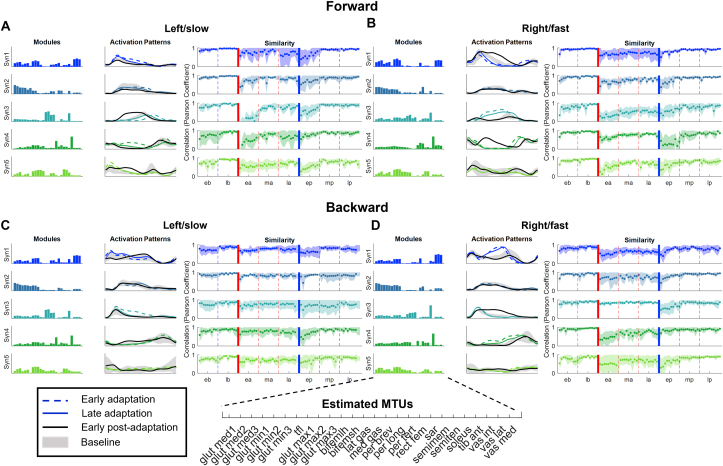
Synergies adaptation. The four panels show the adaptation behaviors for each side (left, slow and right, fast) and each experiment (FW top, BW bottom). Specifically, panel (A) shows left/FW, panel (B) shows right/FW, panel (C) shows left/BW and panel (D) shows right/BW. Each panel shows, from left to right: first plot - the average (across subjects) modules extracted at baseline; second plot - the average (across subjects) activation patterns during specific phases of the experiment (the dashed lines represent the average during all the steps of early adaptation, the solid line the average during late adaptation, the black line the average during early post-adaptation; the gray shaded area represents the average ±standard deviation during baseline); third plot - the average (across subjects) similarity values (calculated using Pearson's correlation coefficient) derived from the average baseline activation pattern of each synergy and the activation pattern of each Step in the different phases of the experiment. Shaded areas represent the standard deviation across subjects. The vertical solid lines represent the transition between baseline and adaptation (red) and between adaptation and post-adaptation (blue). The vertical dashed lines represent the transitions between the different sub-phases of each phase.

On the other side, the synergy activation patterns change substantially during the different phases of the FW and BW experiments (Figure 2), similarly to what we previously observed using a robot-based locomotor adaptation paradigm [17]. In both the FW and BW experiments we found patterns of modulation of the activation patterns that appear to be laterally disjointed, meaning that the two legs adapted their synergies independently. Adaptation to split-belt FW is bilateral, as noticeable from the exponential behaviors in the synergy similarity values observed for all synergies on both sides (Figure 2). In the following, we list the main changes in the synergy activations in order of apparent magnitude. The biggest changes happened in the synergy characterizing forward propulsion (Syn3, Figure 2). At the beginning of the split-belt phase the calf synergy on the fast side presented an increase in intensity (125% compared to the intensity at baseline, Figure S2) over mid-late stance, that was not adapted for, likely because of the increased need for forward propulsion due to the faster belt. The activation of this synergy was also anticipated in the fast leg cycle (-15% of the cycle duration, Figure S2), but the timing was adjusted back to its baseline value towards the end of the split-belt phase. The same synergy on the slow side presented the opposite shift in timing (+20% of the cycle duration, Figure S2), that was also compensated for by the end of the phase. Similar timing changes were observed also in Syn1 and Syn2 (stabilization during early and mid-stance) on both sides, with the fast side presenting an initially anticipated activation profile (about 5% between the two synergies, Figure S2) and the opposite happening on the slow side. We also observed small initial increases in synergy intensity at the beginning of the split-belt phase for Syn4 (swing phase) on both sides (+184% and +96% for the slow and fast side respectively, Figure S2) and Syn1 on the slow side (about +141%, Figure S2), but also, in this case, the initial changes were subsequently adapted for. In summary, split-belt FW induced initial changes in intensity and timing of activation in most synergies that were, nevertheless, mostly compensated for (similarly to the above-discussed changes in symmetry), with the only lasting changes happening on the activation profile of the propelling synergy on the fast leg that presented an increased activation profile during mid-late stance for all the duration of the split-belt phase. At the beginning of the post-adaptation phase, we observed major changes in activation of some of the synergies (e.g. Syn1 and Syn4 fast side, Syn3, slow side) that were, in some but not all cases (e.g. Syn1 fast side), opposite to the changes observed at the beginning of the adaptation phase.

During BW, the neuromuscular adaptation was also bilateral, but with more prominent changes in the recruitment timing of the synergies on the fast side and smaller adjustments in activation intensity on the slow side, compared to FW. In the following, we list the main changes in the synergy activations in order of apparent magnitude. On the fast side, adaptation was characterized by changes in timing and intensity of Syn1 (quadriceps) that represents the recruitment of the muscles mostly responsible for backward propulsion during BW. At the beginning of the split-belt phase, the activation profile of the synergy appeared to be anticipated, (with peak activation happening approximatively 15% earlier during the cycle) and presented an increased intensity during mid-to-terminal stance. Similarly, to what observed for Syn3 in FW, the initial changes that occurred at the beginning of the split-belt phase were only minimally compensated for. Small changes were also observed for Syn4 (tibialis anterior) on the fast side, which presented an anticipated and increased activation (-11% and 68%, respectively) that was mostly compensated for. On the slow side, we observed small changes in all synergies, that were mostly characterized by a small increase in synergy activation intensity (e.g. Syn1, Syn3, and Syn4) rather than timing changes. At the beginning of the post-adaptation period, we observed prominent changes in activation in some of the synergies on both sides (e.g. Syn1 slow, Syn3 fast side) that quickly disappeared. In summary, BW presented smaller initial changes in the synergies compared to FW at the beginning of the exposure to the split-belt condition (i.e. change in speed of one belt), but adaptation was obtained using the same overall strategy of increasing and anticipating the activation of the propelling synergies on the fast side. It should be noted that the synergy adaptation results hold true both for the analysis based on the estimated MTUs (Figure 2) and the same analysis performed on the recorded EMGs (Figure S10).

### 3.3 Symmetry, step-timing, kinetics and kinematics adaptation

**Figure 3:**
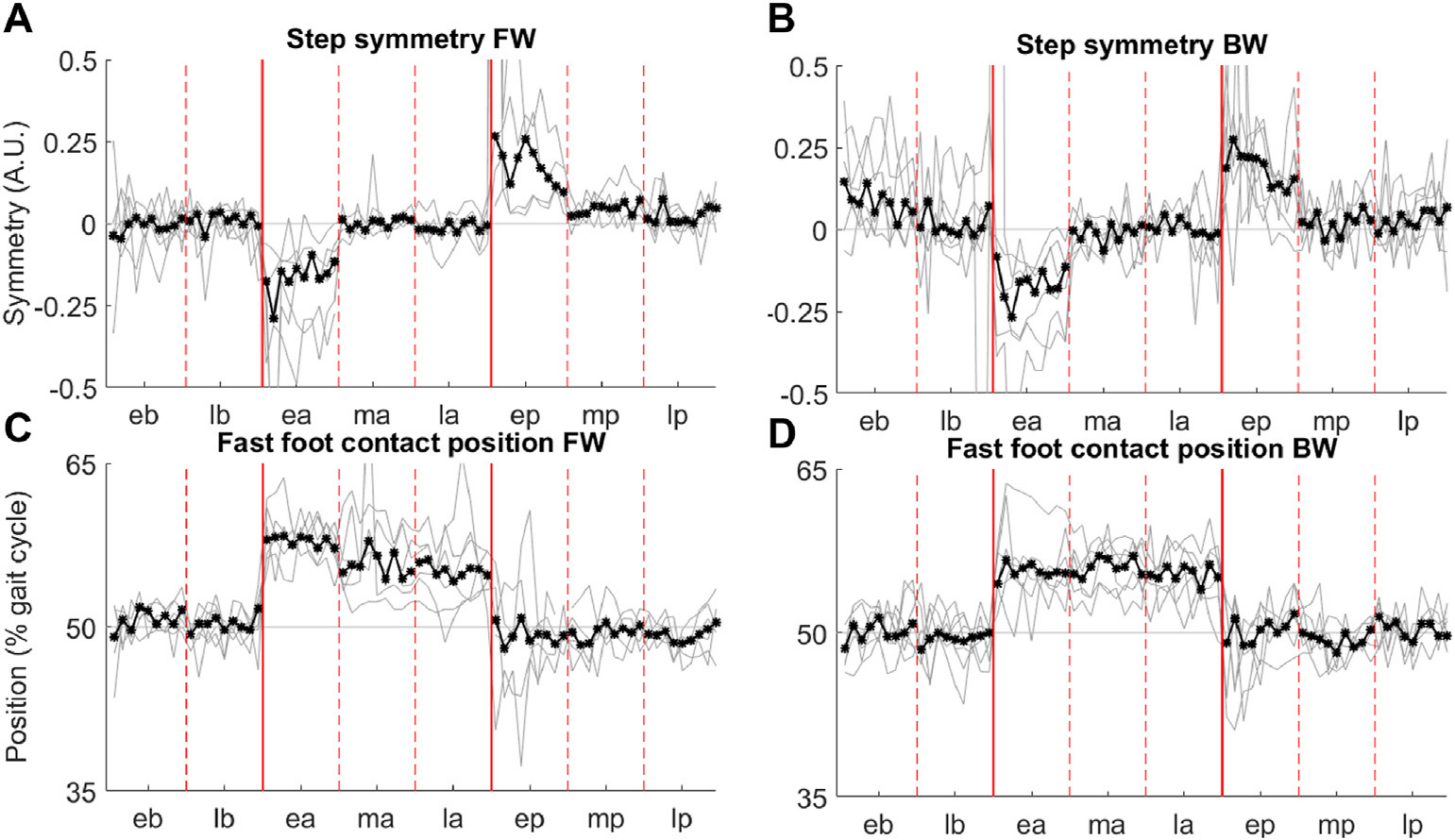
Symmetry and Step timing adaptation. The top two panels (A) and (B) present the results for the step symmetry index calculated over the different phases of both experiments. A value of 0 indicates perfect symmetry between the two legs. The bottom panels (C) and (D) present the results for the timing of the foot contact of the fast limb in the gait cycle of the slow limb expressed as percentage of the gait cycle duration for both experiments. In all four plots the black lines and points represent the average across subjects, the grey lines represent the individual subjects' data. The vertical solid lines represent the transition between different phases (b = baseline, a = adaptation, p = post-adaptation), while the dashed lines represent the transition across sub-phases (e = early, m = middle, l = late) of each phase.

It has been observed that split-belt adaptation is characterized by a restoration, over the course of the split-belt portion of the experiment, of step length symmetry [36]. In both the FW and BW experiments, we observed (Figure 3) a decrease in the step symmetry index equal to about 0.25 at the beginning of the adaptation phase, due, in both cases, to the right limb taking transiently a shorter step on the faster belt while the left limb took longer steps throughout the whole phase [36]. In both experiments the asymmetry was fully adapted for before the beginning of the middle part of adaptation phase. At the beginning of post-adaptation, we observed, once again in both experiments, an aftereffect opposite in direction to the original asymmetry, that was marked by a longer step on the right side and shorter ones on the left side. All these observations are in line with what is typically observed during split-belt treadmill experiments [36, 37, 38]. The changes in symmetry were not accompanied by changes in the relative stepping time of the two legs, here characterized by the timing of foot contact of the fast leg during the slow leg gait cycle. At the beginning of the split-belt phase of both experiments, subjects delayed the heel contact of the fast leg by approximately 7.5%. This delay remained consistent throughout the whole split-belt phase, with only a minimal decrease during the FW experiment. This change was not accompanied by an opposite aftereffect once the belts were set back to the same speed, thus indicating the absence of an adaptation behavior in inter-limb step timing.

Kinematic and kinetic adaptations were mostly characterized by timing and amplitude changes at the ankle joint level on both sides (Figure S3) [39, 40, 41]. Timing adjustments observed at the joints were mirrored between the two limbs in both experiments (although most prominently in the FW experiment), with the faster limb showing an initial pattern characterized by anticipated peaks in the kinematics and kinetics that was compensated through the adaptation phase, with the opposite happening in the slow limb.

### 3.4 Longitudinal stability adaptation

**Figure 4:**
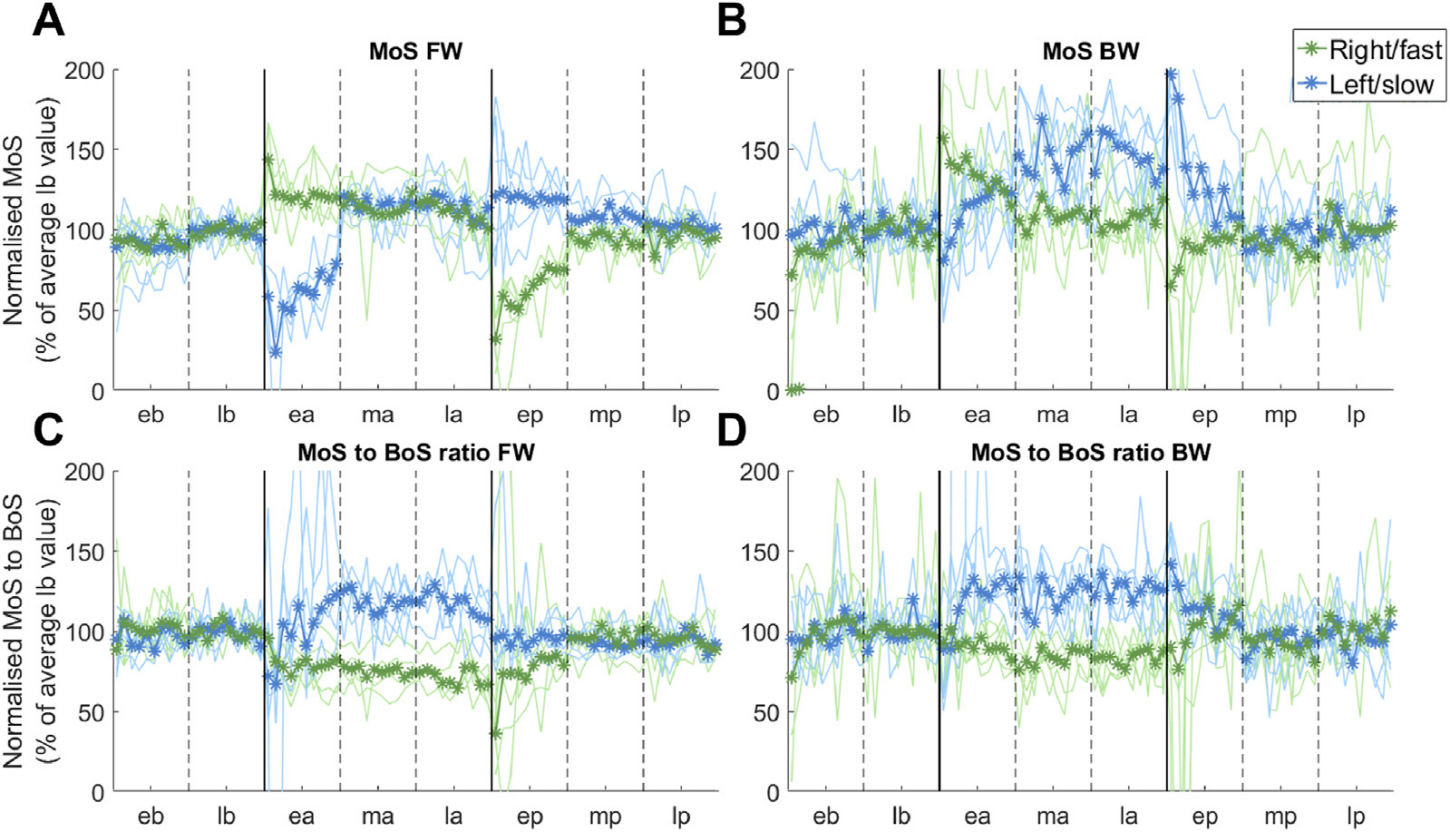
Stability adaptation. The four panels show the values of the margin of stability (MoS, panels A and B) and the margin of stability to base of support ratio (MoS to BoS ratio, panels C and D) for the two experiments (FW panels A and C, BW panels B and D). Each plot shows the values of the parameters expressed as a percentage of their average value at baseline. For each plot, the darker line and points represent the average (across subjects) while the lighter lines represent the values of these parameters for the individual subjects. The blue lines represent the results for the left, slow leg, while the green lines represent the results for the right, fast leg, captured at the time of foot-off. Vertical solid lines represent the transitions between different phases of the experiment (b = baseline, a = adaptation, and p = post-adaptation), while the vertical dashed lines represent the transitions between the different sub-phases of each phase (e = early, m = middle, and l = late).

In a previous work on robot-perturbed locomotion, we provided evidence that stability is the parameter that primarily drives the adaptation process during walking [23]. To test if this hypothesis held for the split-belt treadmill experiments, we evaluated the changes in dynamic stability during both experiments by examining the margin of stability (MoS) [42] parameter in the sagittal plane. This parameter represents the distance between the forward component of the extrapolated center of mass and the boundaries of the base of support. We also calculated a new parameter that is the ratio between the MoS and the size of the base of support (MoS to BoS ratio) that accounts for changes in size in the base of support due to the subject taking shorter/longer steps. Both parameters were calculated at the time of foot-off of each leg (Figure 4, non-normalized results are presented in Table S1).

In the FW experiment, the MoS during the baseline phase was maintained (across subjects on both sides) at an average value of 20 cm (Tab S1), which corresponded to approximately 52% of the BoS. Immediately after introducing the split-belt condition, the MoS increased on the right, fast limb by 40% (Figure 4) and the MoS to BoS ratio decreased by 25% compared to baseline, thus indicating an increase in the size of the BoS. On the left, slow limb, the MoS decreased initially by 42% and the BoS to MoS ratio decreased by 25%, indicating a decrease in the BoS on the slow side. These results show that the initial decrease in gait stability at the beginning of the adaptation phase of the experiments is prominent at the time of left foot-off, when the fast side becomes fully weight-bearing. Then, over the course of the adaptation phase, participants changed their gait stability towards what appears to be a more cautious gait plan. In fact, the MoS parameter presented, on both limbs, values 4%–5% greater than baseline towards the end of the adaptation phase, corresponding to changes in the MoS to BoS ratio parameter of -30% and +17% compared to baseline for the fast and slow legs, respectively. These results indicate that subjects slightly overcompensated the change in MoS by increasing the size of the BoS on the slow side while leaving it unaltered on the fast side (Figure S4).

For both legs, the changes in MoS followed an exponential adaptive behavior, as Step symmetry. At the beginning of the post-adaptation phase we observed an opposite deviation from the baseline behavior on both limbs, namely a steep decrease in the MoS on the fast side, due to the shorter step caused by the adaptation aftereffect, and a slight increase in MoS and BoS on the slow side. This behavior was quickly washed out.

The results for the BW experiment were mostly consistent with the ones for the FW experiment (Figure 4). The MoS for the BW baseline was about 13–14 cm across subjects, equal to about 40% of the BoS, thus we observed a decrease in the MoS during BW when compared to FW. At the beginning of the split-belt phase, subjects increased their MoS on the fast side by about 55%. We observed a small initial decrease in the MoS on the slow side. Over the course of the adaptation phase, the MoS increased by 50% for the slow leg and decreased back to its baseline value for the fast leg. Similarly, the MoS to BoS ratio presented values 10% smaller than for baseline for the fast leg and 30% higher than for baseline for the slow leg. These results indicate that subjects adopted the same strategy as for the FW experiment to compensate for the stability threat posed by the split-belt condition, consisting of adjusting mostly the size of the BoS on the slow limb. The difference compared to the FW experiment is that, in BW, individuals appeared to present a more over-conservative gait towards the end of the split-belt phase. To achieve this, the participants consistently kept their COP closer to the center of the BoS during the split-belt phase, translating in values of MoS on the slow side that were about 40% greater than their correspondent values at baseline. During post-adaptation, the increased values in MoS rapidly returned to the baseline values.

### 3.5 Energy consumption adaptation

**Figure 5:**
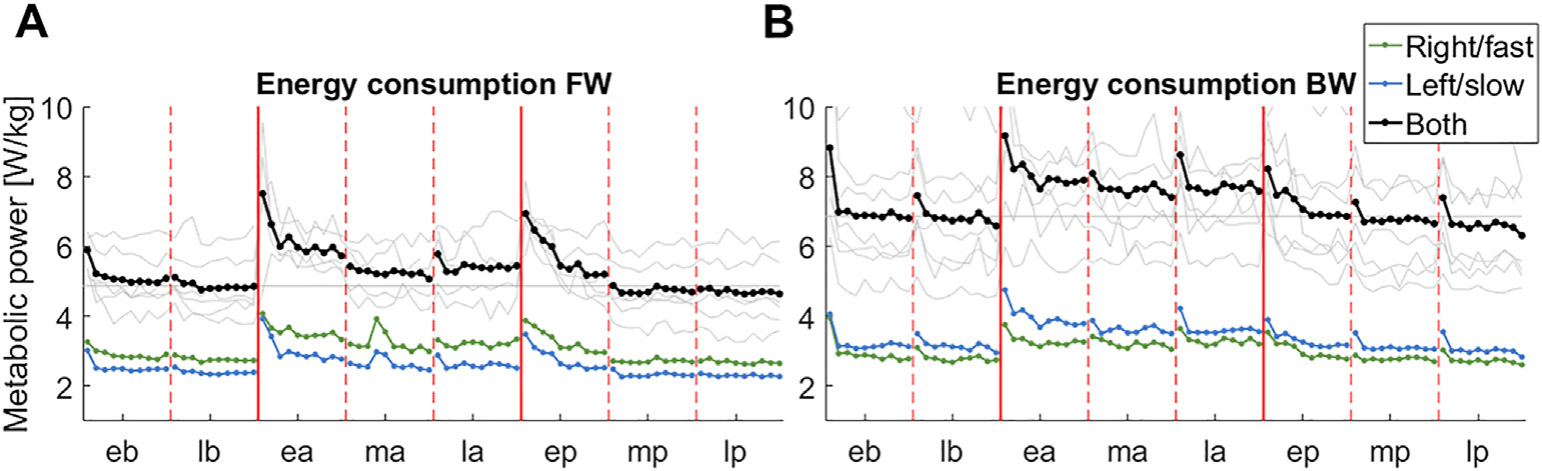
Energy adaptation, raw data. The panels show the estimated metabolic power (expressed in W/kg) through the course of the two experiments (panel A for FW, B for BW). In each plot the black line represents the average (across legs and subjects), the grey faded lines represent the average across legs for each subject, the blue line represents the average (across subjects) for the left, slow leg and the green line represents the average (across subjects) for the right, fast leg. Vertical solid lines represent the transition between the different phases of the experiment (b=baseline, a=adaptation, and p=post-adaptation), while the vertical dashed lines represent the transition between the different sub-phases (e=early, m=middle, and l-late).

**Figure 6:**
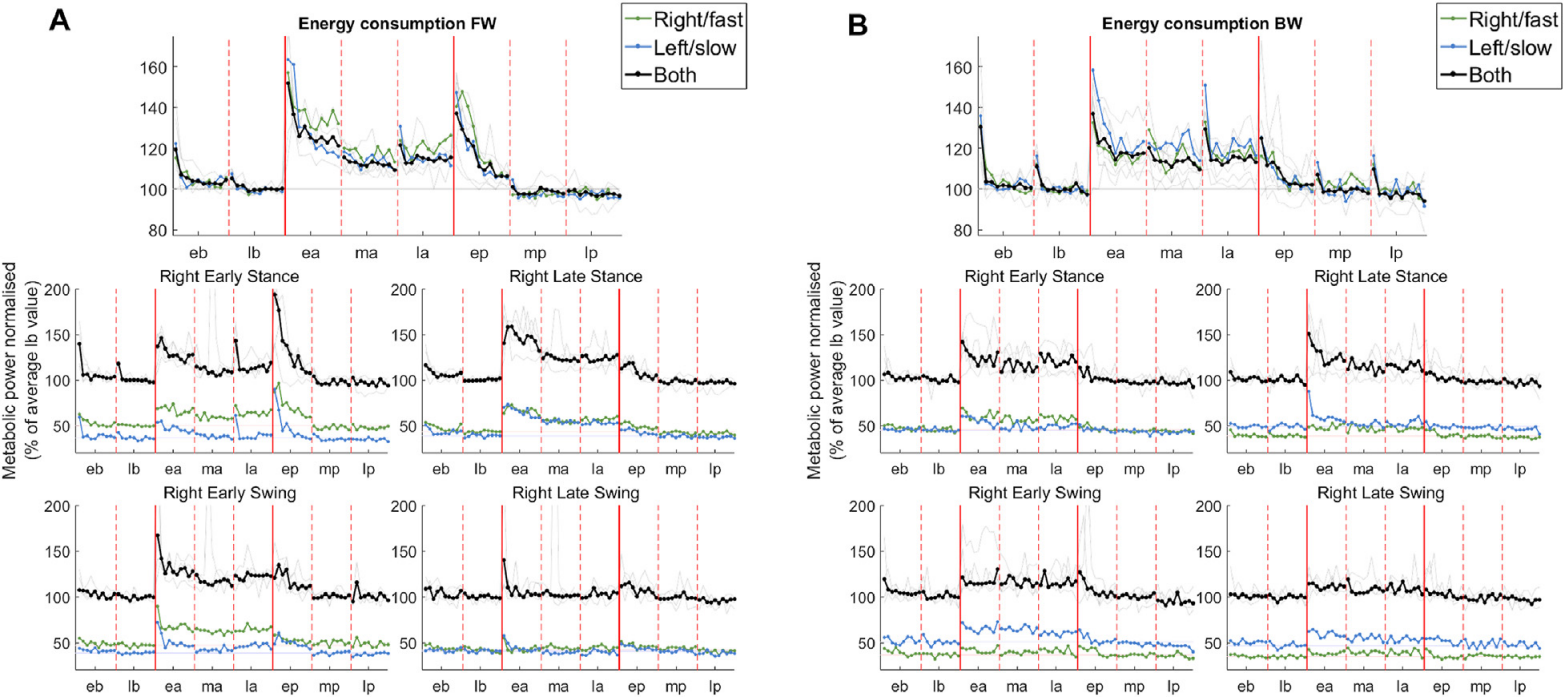
Energy adaptation, normalized and gait phase-specific data. Panel (A) presents the results for the FW experiment, panel (B) for the BW experiment. In each panel, the top plot shows the results for the whole gait cycle, while the other four plots show the results relative to different sub-phases of each gait cycle (early and late phases of stance and swing). Each plot shows the estimated metabolic power (expressed as a percentage of its average value at baseline) through the course of the experiment. In each plot the black line represents the average (across legs and subjects), the grey faded lines represent the average across legs for each subject, the blue line represents the average (across subjects) for the left, slow leg and the green line represents the average (across subjects) for the right, fast leg. Vertical solid lines represent the transition between the different phases of the experiment (b = baseline, a = adaptation, and p = post-adaptation), while the vertical dashed lines represent the transition between the different sub-phases (e = early, m = middle, and l-late).

It has been shown that split-belt walking induces an increase in energy expenditure that is minimized over the course of the adaptation period [43]. Energy consumption is usually estimated from the rate of oxygen consumption and carbon dioxide production using metabolic systems. These systems enable the estimation of the energy consumption of the whole body but have limited time resolution. Here we used the MTU activity (estimated from all the 66 MTUs) to estimate the energy consumption due to the activity of the lower limb muscles during both experiments (Figures 5 and 6) using models linking energy consumption to muscular activity [25, 26] that have been shown to present acceptable agreement with experimental data.

The average estimated lower-limb energy consumption at baseline for the FW condition was equal to 5 W/kg (Figure 5) and was lower than that observed for the BW condition (7 W/kg). The estimated energy consumption for the FW experiment is consistent with the values usually reported for FW based on models, that vary around 3.5–5 W/kg [44, 45]. BW has been shown to be about 20% more energy-expensive [46] than FW, but in our analysis we observed an increase of about 40%. Interestingly, we observed a trend by which, for FW, the energy expenditure was slightly higher on the right side compared to the left side, while we observed the opposite behavior for BW. In both experiments, we observed a steep increase in energy rate at the beginning of the split-belt phase, reaching a peak about 40–50% higher than the baseline energy consumption value (Figures 5 and 6). In the FW experiment, the energy expenditure showed full adaptation during the first third of the adaptation phase towards a new level of about 5.5 W/kg (about 10% more than normal walking). In the BW experiment the energy expenditure also adapted at the beginning of the experiment, reaching a plateau of about 8 W/kg (20% more than baseline) during the first 5 steps of the adaptation phase. At the beginning of the post-adaptation phase, we observed, once again, a sudden increase in the energy expenditure for the FW experiment, reaching a peak about 40% higher than the baseline energy consumption value. Subjects then were able to quickly re-adapt to their baseline level of energy consumption. In the BW condition, we observed a small increase in energy consumption during the post-adaptation phase followed by a fast exponential decrease in energy expenditure back to its baseline value once the belts were set back to the same speed.

To gather additional insights on how the energy consumption adapts throughout the gait cycle, given the additional time resolution that the model-based energy calculation approach enabled, we estimated the energy expenditure relative to four different sub-phases of the gait cycle, specifically to early and late stance and swing (Figure 6). For FW the initial increase in total energy consumption during the split-belt phase was present in all four phases of gait cycle. However, it appeared to be mainly driven by changes in the fast limb in late stance and early swing (corresponding roughly to late swing and early-to-mid stance in the slow limb), and most of the adaptation in the slow limb happened during late swing to midstance (e.g. quadriceps and tibialis anterior). During the late stance phase of the fast leg cycle (corresponding to late swing/early stance of the slow limb), the energy calculated for the two legs followed the same adaptation pattern. In late swing, we only observed a peak in the first Step of split-belt walking and then the energy expenditure quickly returned to its baseline value, thus indicating a transient effect. All in all, the energy changes in FW split-belt adaptation appeared to be mostly driven by the propulsion phase of the fast leg and the stabilization of the slow leg after landing, as also expected from the changes observed in the MTU synergy activations.

In the BW experiment, the adaptive, exponential changes in energy rate are mostly observed during the early and late stance phases of the gait cycle of the fast limb (Figure 6). In early stance (corresponding to late stance/early swing on the slow side) changes were mostly observed in the fast leg, primarily due to the changes observed in the hip flexion synergy. During late stance (corresponding to late swing/early stance on the slow side) the increase in energy expenditure was mostly due to the slow leg, in parallel with the increase in activation observed in the calf synergy on that side. During the swing phase of the fast limb, we observed a Step-like increase of about 10% in energy expenditure that was mainly driven by changes in energy expenditure happening on the slow limb during the stance phase. The initial changes in energy consumption in both experiments appeared to be due to the initial stability-driven response to the perturbation, while the subsequent adaptation provided an optimization of the economy of the emerging walking pattern.

## 4 Discussion

The aim of the study was to characterize the neurophysiological, motor control, and adaptation differences and similarities between FW and BW using a multilevel, model-based analysis of the two walking directions during split-belt adaptation. Neuromechanical models have become a useful tool for analyzing the processes behind the generation of gait behaviors and their alterations [44, 47, 48]. Here we utilized modeling to characterize the neuromuscular and energetic correlates of FW and BW during split-belt adaptation by estimating the activity and energy consumption of a comprehensive number of lower limb muscles. We found, in apparent discordance with previous literature [8, 19, 20], that FW and BW are obtained by employing the same synergy modules recruited with similar or different temporal activations between the two directions depending on the role of the muscles in FW and BW. We also found that adaptation is achieved, for both walking directions, by modulating the activation of these low-level muscle synergy modules, which remain unaltered in response to the perturbation (i.e. in response to the change in speed of one of the treadmill belts). Patterns of adaptation were consistent between the two directions, which displayed changes in stability and energy consumption in response to the perturbation. Specifically, we found that the adaptation process is well explained by an initial exaggerated response to the perturbation aiming at maintaining stability that is optimized energetically over the course of the adaptation period. In contrast to FW, BW is not a commonly used pattern of locomotion and cannot rely on visual input for long and short term stability planning [49]. For these reasons, BW is likely slightly less stable [50], less trained and automated [51] and less energy-efficient than FW [46]. Nevertheless, our results show that the adaptation processes during FW and BW split-belt walking are consistent, as they are marked by the same high-level response strategy and criteria for execution plan. This observation indicates that both walking directions trigger high-level adaptation processes that appear to be independent on the walking direction.

### 4.1 Neural control of FW and BW

A growing body of literature in animal models and humans has shown that locomotion patterns can be described, at the neuromuscular level, by the repetitive recruitment of remarkably stable muscular muscle synergy modules. Whether in humans these blocks represent physiological structures encoded in the spinal cord since birth [1] or arise from physiological constraints [52] is still unclear and much debated in current literature. Animal models have shown that synergies appear to be encoded in the spinal cord [11] and to be accessible by both descending and reflexive drives [53], possibly to regulate the concert of activity of the redundant number of muscles that contribute to the performance of different motor tasks [54] and to satisfy the biomechanical requirements of each task and hence generate approach forces at the joint level [55]. In humans, these modules have been shown to be consolidated throughout the development period [1, 2], shared across different tasks [14, 15], consistent during the response to perturbations [16, 17, 56] unless the function of one or more muscles in the task is altered [28] and possibly, as in animals, accessible by both reflexes and descending drives [17]. Nevertheless, the same motor modules observed in experimental studies can also arise from biomechanical simulations [52] suggesting that biomechanical constraints can be enough to elicit the most commonly observed muscle modules during human locomotion, but not during more complex movements (e.g. upper limb movements [57]).

Given the observation, made in several seminal studies [4, 8], that BW can be described (visually, kinematically and kinetically), as a time-reversal of FW, can the associated neural control be explained, at least partially, by different activations of the same muscle modules? Our results appear to confirm this hypothesis, as we observed identical MTU synergy modules for FW and BW (Figure 1). The EMG activity during FW and BW is generally different [8] and cannot be explained by a simple reversal of muscle activation patterns. This should not be expected given the complex non-linear relationship between muscle activations and biomechanics and the different function that some muscle groups have during FW versus BW. The few studies that reported muscle synergies analyses of lower limb muscles showed that these two tasks are obtained by recruiting different synergies [8, 19, 20]. On the other hand, our results - while confirming that the muscle activity patterns for FW and BW are different (Figure S5) - revealed that identical muscle modules (Figure 1 and Figure S6) underly the generation of movement for the two tasks, and that these modules are activated via similar and different activation patterns, depending on the role of each synergy.

The difference between ours and previous results can be explained by methodological factors. Synergy analysis has been shown to be critically dependent on the choice of the muscles analyzed [58], on the pre-processing of the EMG signals [59] and on the algorithm used for the decomposition [60]. Two of the mentioned studies that showed differences in FW and BW synergy modules employed PCA to study the timing of the activation of different muscle groups [8], and in one of these two studies the authors performed the decomposition by fitting the activity of the muscles using baseline temporal pulses [19]. PCA and NMF employ basis vectors of different nature, hence the results obtained using these two algorithms cannot be compared directly [61]. Additionally, PCA has been found to be performing worse than non-negative matrix factorization (NMF) [62] algorithms in uncovering the shape of the synergy modules [60], and, due to the orthogonality of the basis vectors, may lead to negative weights of muscular activation that are uninterpretable physiologically [61]. A more recent paper that employed NMF for analyzing FW and BW synergies selected a different number of synergies for FW and BW, based on the quality of the reconstruction of the EMG envelopes of the whole dataset and the individual muscles [63]. Herein, instead, we fixed the number of synergies to 5 for all subjects and both tasks. We selected 5 modules because this number of synergies yields an average (across participants) reconstruction level R^2^ above 0.95 during the extraction of the reference synergies used for the semi-fixed synergy analysis (see *Methods*), a reconstruction level that is acceptable by literature standards. We used this number both for the MTUs activations estimated using the neuromuscular model (Figure 1) and for the recorded muscles alone (Figure S6), regardless of the quality of reconstruction.

This choice is consistent with the literature that shows that 4 to 5 synergies can be used to describe locomotion and the adaptation processes [17, 64, 65, 66]. We found that reconstruction levels, for both datasets, are comparable between the two conditions and acceptable by literature standards. Starting from the observation that any criterion for selecting the number of synergies reconstructing a task is inherently arbitrary, enforcing the same number of synergies between two tasks assures that the same motor modules can be identified if they are employed in both tasks. In fact, if the two tasks share *n* synergies and one of them employs *m* additional unshared synergies, enforcing the same number of synergies *n* would result in the second task presenting synergies with a composition consisting of merged versions of the shared and unshared ones. On the contrary, if both tasks were reconstructed using *n*+*m* synergies, the task characterized by fewer synergies would present synergies that are fractioned versions of the shared pool [67]. It follows that, if two tasks, as in our case, are reconstructed using the same number of synergies and the composition of the modules is identical, barring acceptable levels of reconstruction for both tasks, the two tasks share the same modules and the same dimensionality. Finally, while we performed our analysis focusing on data from the muscles of the hip, knee and ankle joints the two most recent studies that found different modules for FW and BW performed their analyses on a bigger pool of muscles comprising foot, back and abdominal muscles. Hence, it is possible that we were able to identify common synergy modules because our muscle pool almost exclusively represents the contribution of the lower limb muscles to locomotion on the sagittal plane (although we also analyzed muscles contributing to hip adduction/abduction) and that different synergy modules might be utilized to control the degrees of freedom acting on the other planes. It is then possible that if we were to include more muscles acting on different joints and degrees of freedom, some of the synergies between FW and BW would differ.

Interestingly, our results appear to be in some accordance with a recent model-based study [18] that found that most muscles have a time-reversed activation in their contribution to horizontal acceleration during FW and BW, while their contribution to vertical acceleration (e.g. response to gravity) is the same between BW and FW. Although the authors of that work did not execute a muscle synergies analysis and we did not analyze the contribution of each synergy module to the different acceleration components, those results appear to imply that the muscle synergies may be similar between the two tasks and may present similar or time-reversed activation patterns.

### 4.2 Neuromechanical patterns of adaptation during FW and BW

As in previous studies with focus on both upper and lower limbs, from our and other groups, we found that adaptation was completely described by a modification of the activation of the same synergy modules, which changed minimally in response to the perturbation [17, 27, 29, 31, 56, 68]. The temporal and amplitude changes in the activation of the synergy modules were mostly related to changes in propulsion needs during both experiments, as also reflected in the kinematics and kinetics of locomotion. In the FW experiment, during the perturbation period, the activation of the plantar flexors in the fast limb started earlier during the stance phase of the fast leg and presented an increased amplitude to provide a stronger push off. This was mirrored by the increased and prolonged ankle moment during the stance phase (Figure S3). The opposite timing response, not accompanied by changes in the amplitude of activation, was observed in the plantar flexors on the contralateral side. Exponential adaptation patterns were observed, on both sides, in response to the initial change in synergy activation timing, while the amplitude change on the fast side was not adapted for, mostly because the fast limb required a stronger push-off throughout the split-belt adaptation period. The timing changes in the calf synergies on the two sides translated in opposite timing effects in the joint angles (Figures 3, S2, and S3), that were partially compensated for during the experiment. Our results on the temporal activation of the synergies are consistent with those observed in a previous study on muscle synergies during a split-belt experiment, in which the authors observed matching anticipation in both the slow and fast plantar flexor synergies when normalizing the gait cycle to the fast limb Step cycle (Figure S2) [65]. The temporal adjustments that we observed are bigger presumably due to the higher split-belt ratio employed in our study. We also observed an increased activity of the TA synergy of the slow leg during the stance phase, that adapted throughout the experiment. This is expected to help stabilizing the leg during the longer single support [40, 41] and slowing down the center of mass [50].

Our work is the first to report how neuromuscular control adapts during BW split-belt. Overall, the effects observed in the kinematics and kinetics of locomotion were smaller for BW than for FW, consistently with previous results [68]. During BW, the transition between stance and swing is not driven by a push-off but rather by the lifting of the leading limb. The ankle joint still contributes to propulsion but its main role becomes shock absorption [69]. Thus, the timing and synchronization of gait, and therefore, the adaptation process, appear to be controlled mostly by the hip joint. For this reason, the main changes at the neuromuscular level are observed, on the fast side, in the synergy controlling the activation of the hip flexors instead of the one controlling the plantar flexors. In the hip flexor synergy, we observed an anticipation of the activation peak during stance that did not change during the split-belt perturbation period. We also observed an anticipated and increased activation of the dorsiflexor synergy around midstance, likely aiming at slowing down the displacement of the center of mass in the sagittal plane [18, 50]. The response to this synergy was partially adapted for throughout the course of the experiment. On the slow side, we observed small initial changes in the amplitude of activation of the plantar flexor synergy during the stance phase. As this synergy mainly contributes to shock absorption, this initial increase in activation, that was exponentially adapted for, could be an over-conservative response to the initial stability threat represented by the anticipated timing of foot-off on the fast side during early stance on the slow side. Overall, the neuromuscular changes that we observed during BW were more prominent on the fast side. This is in contrast with what we observed in FW, where changes were observed on both sides. This result is in line with our previous observation that the laterality of adaptation is task-dependent [17] and with the hypothesis that central pattern generators adapt independently between the two legs [21, 28].

### 4.3 Adaptation processes and bi-level control of gait

Choi and Bastian have shown that BW does not wash out adaptations to FW split-belt and vice-versa, suggesting that the two walking directions are controlled by independent functional networks [21]. Combining this observation with the results herein reported allows us to suggest that the neural circuits driving the adaptation processes for FW and BW affect the control of locomotion at a higher level than the level at which the synergy modules are recruited, as the latter are completely unaffected by the adaptation process. Our results, when taken together with previous literature, appear, once again, to hint to the presence of a two-level structure controlling locomotion [9, 10], where the higher level encodes the timing of the activation of the motoneuron pools, while the lower level encodes the muscle co-contraction patterns that regulate the relative activation of different muscle groups and it is accessible by both descending drives and reflex pathways [53].

The lack of transfer between FW and BW split-belt walking could then be explained by the presence of distinct high-level timing control circuits that are involved in the control of movement for the two walking directions. Nevertheless, although in adaptation paradigms such as split-belt treadmill and moving platform there is no transfer or interference between FW and BW adaptations [21], a previous study has found that podokinetic after-rotations (PKARs) do show transfer between the two walking directions [22]. PKARs are characterized by a curved walking trajectory after a training period walking on a rotating treadmill. Earhart and colleagues showed that performing podokinetic adaptation training in FW results in a curved walking trajectory during BW. PKARs depend mostly on somatosensory information on the relative rotation of the trunk and feet [22, 70], that changes during training on the rotating platform. If FW and BW are controlled by activating the same low-level synergy-formation circuits, but high-level activation-timing control of these circuits is completely independent between the two walking directions [21], the transfer of PKARs between FW and BW may depend on a somatosensory-input dependent re-organization of the relative rotation of the trunk with respect to the feet [71] that is either realized independently from the activation pattern formation circuits or, less likely, implemented at the level of the synergy-formation circuits.

### 4.4 Bilaterality of stability adaptation

Previous works showed the importance of stability in the gait adaptation process [23, 72, 73]. Here we estimated changes in longitudinal stability using the margin of stability (MoS), a predictor of the ability to recover from a balance threat [42, 74, 75]. We showed that split-belt walking poses, in both FW and BW walking, an initial threat to stability characterized by a decrease in the longitudinal MoS at the time of foot-off on the slow side due to the decrease in the base of support (BoS) [76]*.* The MoS at foot-off is a key metric of dynamic stability [77, 78]. The adaptation process leads, in both walking conditions, to a substantial change in MoS value thus indicating a stability adaptation that is obtained by increasing the Step length on the perturbed side, which is a known response to stability perturbations experienced during split-belt walking [36, 65]. The analysis of the MoS to BoS ratio shows that in both walking conditions stability is restored by increasing the length of the step on the fast side (Figures 4 and S4). Stability adaptation is hence, at least functionally, a bilateral process, as also shown by recent work by Buurke et al. that investigated stability in the mediolateral direction during a split-belt adaptation experiment and demonstrated that MoS in the mediolateral direction depends on the interlimb coordination [73]. Herein, we show that an initial decrease in MoS at the time of foot-off of the slow limb is compensated by increasing the BoS on the fast side. While no work in literature so far has analyzed longitudinal stability during BW split-belt, a few studies have done that for FW split-belt [79, 80]. Our results are in agreement with what shown by previous work analyzing the average, across-legs, MoS during FW (see Figure S8A). Our results also agree with what shown, for the fast side, in a leg-specific analysis of longitudinal stability, when estimating the MoS at initial contact (see Figure S8B). However, there is a discrepancy between these and ours results on the slow side, where, at initial contact, we observed a behavior that is similar between the two legs, while Park and Finley observed a step-like decrease in MoS that was maintained through all the adaptation period. Although the reason for this difference is not clear, and may depend from methodological differences in the calculation of the MoS, it needs to be pointed out that these studies used a 3:1 belt ratio during the adaptation period, as opposed to the 2:1 ratio we used in this study. It is possible that this experimental difference may have caused the observed differences in the results of the MoS and BoS analyses. In our analyses of the adaptation period, we observed that participants, towards the end of the adaptation phase, overcompensated for the initial disruption in stability, thus exhibiting what appears to be a more cautious gait. However, our analyses cannot explain whether this overcompensation is a byproduct of the adaptation process or an emerging independent behavior.

### 4.5 Adaptation in muscular energy expenditure

Minimizing energy expenditure is one of the controlled task variables during gait [81] and it is also one of the principles underlying locomotor adaptations [43, 82]. Previous work has shown that energy expenditure minimization during split-belt treadmill walking is due to a change in lower limb contraction dynamics and follows a timing that is correlated with the timing of the recovery of step symmetry [43]. Subsequent studies have demonstrated that the reduction in muscle power during split-belt walking depends on the subject learning to take advantage of the work done by the treadmill [83]. Although asymmetric gait is inherently more energy expensive than symmetric gait, it is still not clear if this effect applies to split-belt treadmill adaptation, as the energy increase that we and others observed at the beginning of the split-belt phase of the experiment appears to be caused primarily by the initial neuromuscular response to the perturbation, that happens mostly during stance (Figures 5 and 6). This response appears to be correlated with increased levels of muscle co-contraction likely aimed to address stabilization and increased propulsion needs. Both these changes are progressively optimized over the course of the experiment. Moreover, recent studies have shown that there is not a direct temporal relationship between the reduction in metabolic power during the adaptation period and the return to symmetric gait [72]. These observations suggest that the main cause of the observed increase in energy consumption in response to the perturbation is the non-optimality of the initial, possibly stereotypical (as suggested by the after-effects we observed in the synergies) neuromuscular response, rather than the asymmetry per se. This response is characterized by an increase in ankle power generation of the fast leg that is due to the work done by the fast leg on the belt during the propulsion as a direct response to the stability threat [84].

In our work, we have seen that the overall metabolic cost of transport undergoes an adaptation process that is associated with a noticeable aftereffect. That is different from what reported in previous studies on metabolic changes during split-belt walking, where aftereffects were not evident [43, 72]. Moreover, we showed adaptation in energy consumption that brought energy expenditure close to baseline values towards the end of the split-belt phase of the experiments, whereas other studies have shown that adaptation, while reducing energy expenditure compared to the initial response to the perturbation, leads to levels of metabolic cost that are significantly higher than the baseline ones [43], mostly, in this case, due to residual asymmetry. These differences between ours and previous results likely depends on how energy expenditure was estimated in our work.

In previous studies, full-body energy expenditure was estimated from measures of oxygen uptake. In our study, all energy-related analyses were based on muscle expenditure models [45]. Thus, previous literature reports whole-body energy expenditure during the adaptation process, whereas the energy consumption data discussed in this manuscript is relative only to the muscles included in our model and does not account for other muscles or contingent processes that may increase energy expenditure. This is a limitation of our study that restricts the comparability of our results with previous literature analyzing energy consumption during locomotor adaptations.

Nevertheless, as previously reported, changes in metabolic power during split-belt walking were driven mostly by changes in muscle activity [43]. Hence, studies that estimated energy expenditure during split-belt walking using metabolimeters may capture adaptation-related processes that are not directly related to the activity of the lower limbs, that our setup cannot capture. In our results, we showed that energy adaptation happened, in both experiments, mostly during the stance phase of the fast leg, likely driven by the down-regulation of the initial amplitude increase in the activity of some of the muscles in both the slow and fast legs. The timing of adaptation appeared to be similar to the timing observed for the stability adaptation, but generally faster than that observed using metabolimeters [72]. All in all, the energy adaptation and after-effect that we observed closely resembles the changes in overall amplitude of the 16 recorded EMGs (Figure S9, supplementary material). It needs to be noted that, while the values of overall energy consumption that we estimated using our modeling approach agree with experimental results for FW, they appear to be higher for BW. It cannot be clarified from our data whether this discrepancy is due to limitations in the modeling approach or other reasons.

### 4.6 Limitations

The study herein presented is affected by a few limitations. The main one is the limited sample that was examined. Split-belt treadmill adaptation is a well-known phenomenon that is remarkably consistent across individuals. We believe that adding more participants to the study would most likely not alter the results we obtained on adaptation and stability. From the muscle synergies perspective, the inter-subject variability observed in most locomotion studies is often mostly due to the difference in number of synergies that are identified to better reconstruct the muscular activity of each participant [63]. Here, by setting the same number of synergies across subjects and tasks, we limited that variability while obtaining levels of reconstructions that were above the often-used threshold of R^2^>0.95 and we obtained synergy modules that are remarkably similar across individuals and conditions (Figure S7). Another limitation is that, in our modeling approach, we used 16 recorded channels to estimate a total of 66 MTUs. The ratio between the estimated and recorded data is thus not optimal and higher than what done previously in similar studies [24, 85]. The analysis of residuals, marker errors and joint moments (see *Model Evaluation* in *Methods*) reflects the ability of the MTU-driven model to predict accurate kinematics and kinetics. However, it cannot assure that the estimated activity of the MTUs is fully congruent with the real one. Further studies employing more recorded EMGs are needed to confirm our results.

## 5 Conclusions

The results herein presented show that FW and BW are seemingly controlled by differentially activating the same low-level modules encoding muscle coordination. Adaptation is achieved by modulating the activation of these modules. In both experiments, walking with the belts running at different speeds caused a sudden decrease in stability that was swiftly compensated (FW) or over-compensated (BW) for by adjusting step length on the side of the faster belt. Our results suggest that the energy expenditure changes observed at the beginning of the adaptation phase were a by-product of the energetically sub-optimal reflexive responses employed to facilitate stabilization [84] via the mechanical work done by muscles, that drive the energy changes [43]. The active work was reduced during the split-belt portion of the experiments (i.e. when the two belts of the treadmill moved at different speeds) so as to optimize energy expenditure [83] by leveraging the work done by the treadmill. Our results indicate that the primary mechanism driving adaptation is stability over energy minimization [23, 72].

## Declarations

### Author contribution statement

Magdalena Zych: Analyzed and interpreted the data; Wrote the paper.

Annalisa Cannariato: Performed the experiments; Analyzed and interpreted the data.

Paolo Bonato: Conceived and designed the experiments; Wrote the paper.

Giacomo Severini: Conceived and designed the experiments; Performed the experiments; Analyzed and interpreted the data; Wrote the paper.

### Funding statement

Magdalena Zych was supported by the 10.13039/501100002081Irish Research Council (GOIPG/2018/2987).

### Data availability statement

Data will be made available on request.

### Declaration of interests statement

The authors have no financial interests related to the content of this manuscript.

### Additional disclosures

Paolo Bonato has received grant support from the 10.13039/100000968American Heart Association, the 10.13039/100000005Department of Defense, the 10.13039/100000864Michael J. Fox Foundation, the 10.13039/100000002National Institutes of Health (NIH), the 10.13039/100000001National Science Foundation (NSF), and the Peabody Foundation including sub-awards on NIH and NSF SBIR grants from Barrett Technology (Newton MA), BioSensics (Watertown MA) and Veristride (Salt Lake City UT). Paolo Bonato also received grant support from Emerge Diagnostics (Carlsbad CA), MC10 (Lexington MA), Mitsui Chemicals (Tokyo Japan), 10.13039/100004319Pfizer (New York City NY), Shimmer Research (Dublin Ireland), and SynPhNe (Singapore). Paolo Bonato serves in an advisory role the Michael J Fox Foundation, the NIH-funded Center for Translation of Rehabilitation Engineering Advances and Technology, and the NIH-funded New England Pediatric Device Consortium. Paolo Bonato also serves on the Scientific Advisory Boards of Hocoma AG (Zurich Switzerland), Trexo (Toronto Canada), and ABLE Human Motion (Barcelona, Spain) in an uncompensated role. Giacomo Severini has received grant support (as Principal Investigator) from the EU (H2020) and 10.13039/501100001588Enterprise Ireland. He is Funded Investigator in the Insight Centre for Data Analytics funded by 10.13039/501100001602Science Foundation Ireland.

### Additional information

No additional information is available for this paper.

## Appendix A Supplementary data

Zych_SplitBelt_Supplementary_material_Final_Revision_20_07
