# Supplementary material for: Forward and backward walking share the same motor modules and locomotor adaptation strategies
Source: Heliyon. 2021 Aug 23;7(8):e07864. doi: 10.1016/j.heliyon.2021.e07864 (PMC8405989; doi:10.1016/j.heliyon.2021.e07864)
Supplement: Supplementary file 1 — Zych_SplitBelt_Supplementary_material_Final_Revision_20_07 [file mmc1.pdf]

Forward and backward walking share the same motor modules and locomotor adaptation strategies

Magdalena Zych[1], Annalisa Cannariato[2], Paolo Bonato[2,3], Giacomo Severini[1,2,4*]

[1] School of Electrical and Electronic Engineering, University College Dublin, Belfield, Dublin, Ireland

[2] Department of Physical Medicine & Rehabilitation, Harvard Medical School, Spaulding Rehabilitation Hospital, 300 First Ave, Charlestown, Boston, MA 02129, USA

[3] Wyss Institute for Biologically Inspired Engineering, Harvard University, Boston, MA, USA

[4] Centre for Biomedical Engineering, University College Dublin, Dublin, Ireland.

* Corresponding author

Supplementary material

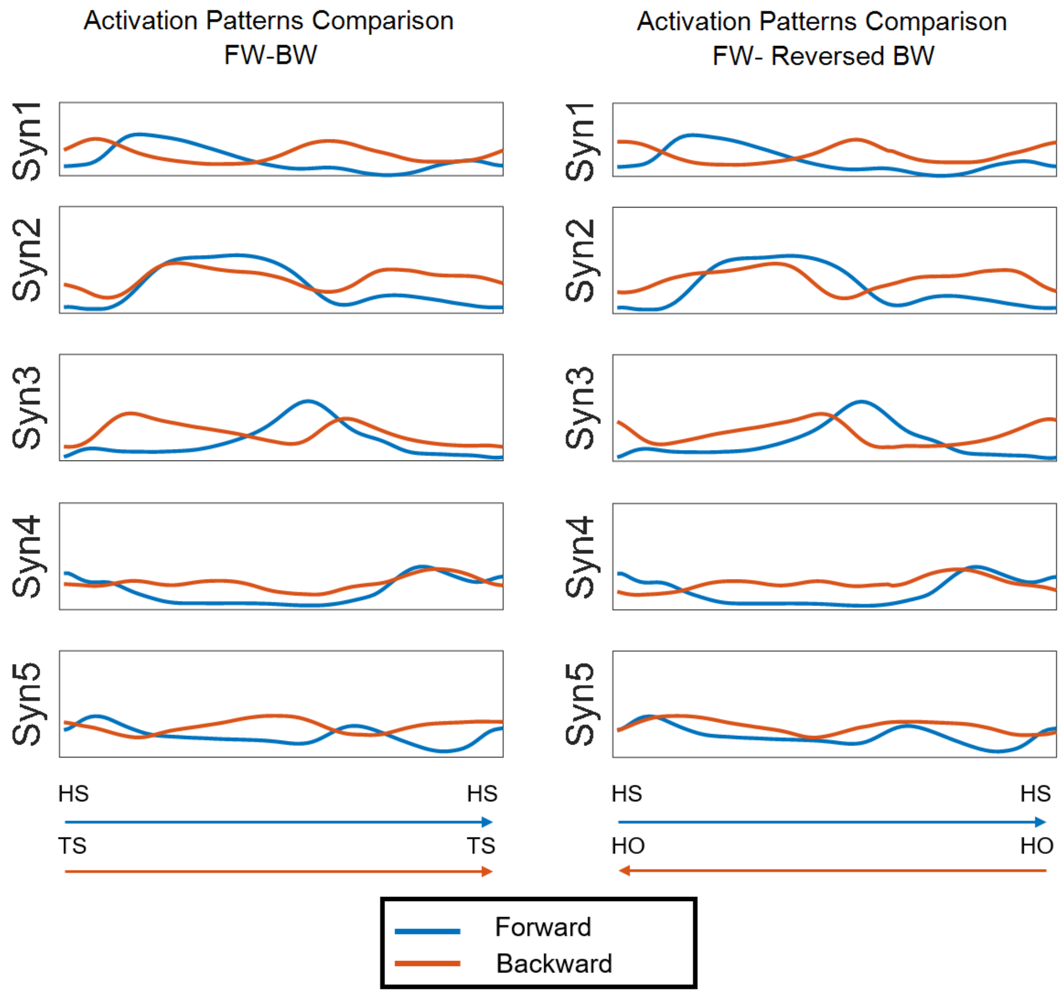

Fig. S1 Synergies activation patterns comparison between FW and BW (normal and reversed). The left column presents the comparison between the average (across subjects and steps) activation patterns at baseline for FW (blue) and BW (red). For FW, the activation patterns are plotted from heel-strike (HS) to heel-strike, while for BW, the activation patterns are plotted from toe-strike (TS) to toe-strike. In this way, we compare stance and swing phase of FW with stance and swing phase for BW. The right column presents the same comparison done after reversing the BW activation patterns. For FW, the activation patterns are plotted from heel-strike to heel-strike, while for the reversed of BW, the activation patterns are plotted from heel-off (HO) to heel-off, in reverse. Also in this case, we compare stance and swing phase of FW with stance and swing phase for the reverse of BW.

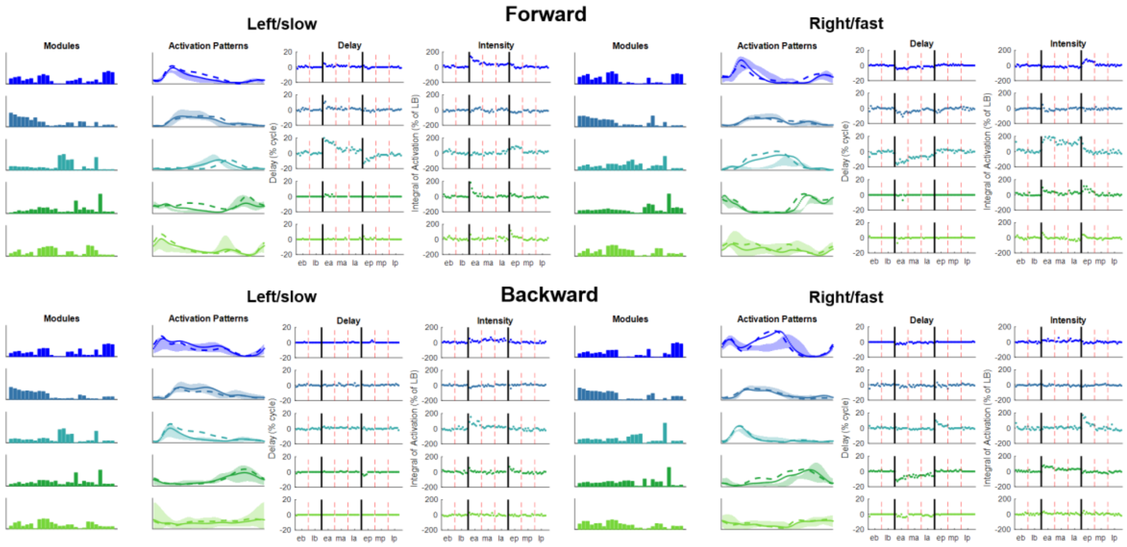

Fig. S2 Synergies adaptation, changes in intensity and timing of the activation patterns. The four panels of the plot present the adaptation behaviors for each side (left, slow and right, fast) and each experiment (FW top, BW bottom). In each panel are presented, from left to right, the average (across subjects) modules extracted at BL and used in the semi-fixed synergies extraction; the average (across subjects) activation patterns through specific phases of the experiment (dashed line represent the average during all the steps of early adaptation, the solid line the average during late adaptation, the shaded area represents the average ± standard deviation during baseline); the average (across subjects) delay (calculated as the position of the maximum of the cross-correlation function) between the average baseline activation pattern of each synergy and the activation pattern of each step in the different phases of the experiment; the changes in activation pattern intensity, calculated as the root mean squared value of the activation pattern and expressed as percentage of the average value at baseline. Vertical solid lines represent the transition between baseline and adaptation and adaptation and post-adaptation. Vertical dashed lines represent the transition between the different sub-phases of each phase.

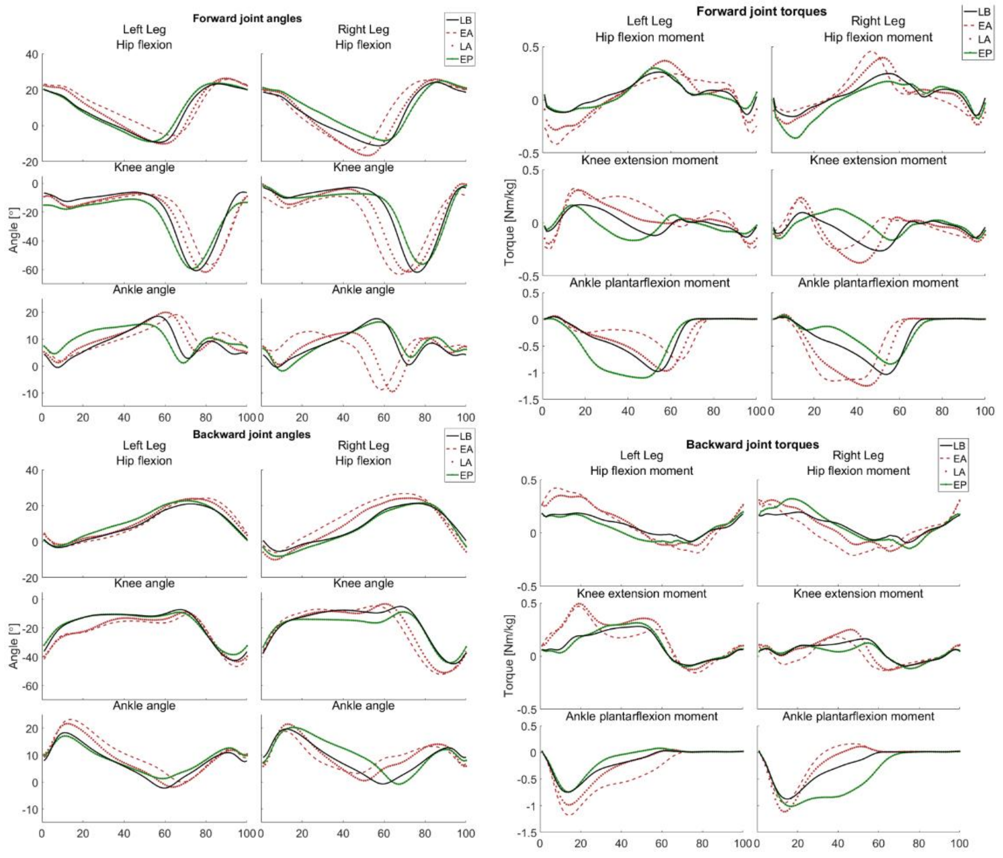

Fig. S3 Joint kinematics and kinetics. The plots present the average (across subjects and steps) joint kinematics (left) and kinetics (right) for the FW (top plots) and BW (bottom plots) experiments. In each plot the solid black line represent the average during late baseline (LB), the dashed red line represent the average during early adaptation (EA), the dotted red line represents the average during late adaptation (LA) an the green line represents the average during early post-adaptation (EP). The x-axis is expressed as percentage of the gait cycle from heel-strike to heel-strike for FW and from toe-strike to toe-strike for BW.

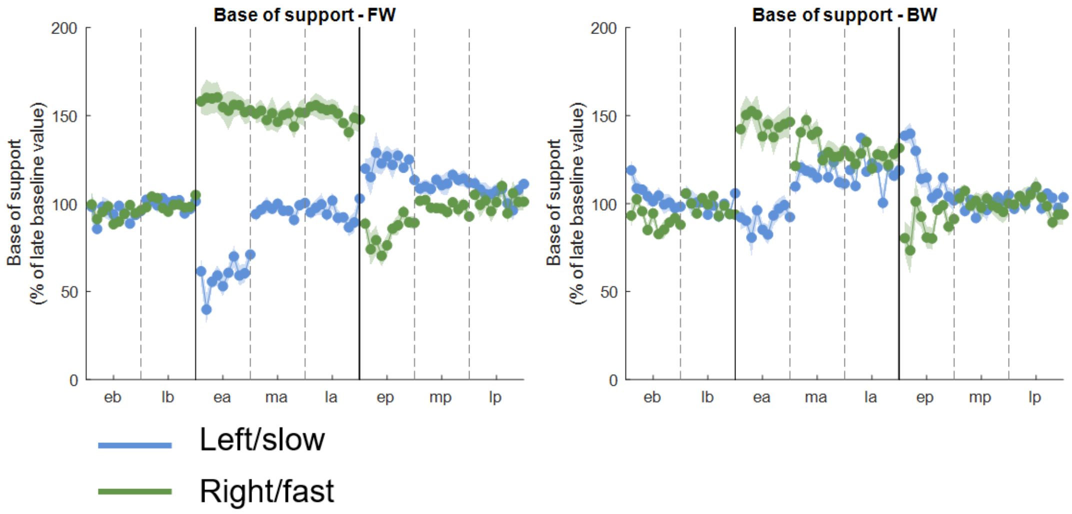

Fig S4. Base of support at toes off. The two plots present the size of the base of support calculated at toe-off for the FW (left) and BW (right) experiments. In both plots, the size the base of support is expressed as a percentage of the average baseline value. Blue lines represent the average (across subjects) values for the left, slow, leg, the green lines represent the average (across subjects) values for the right, fast, leg. Vertical solid lines represent the transition between baseline and adaptation and adaptation and post-adaptation. Vertical dashed lines represent the transition between the different sub-phases of each phase.

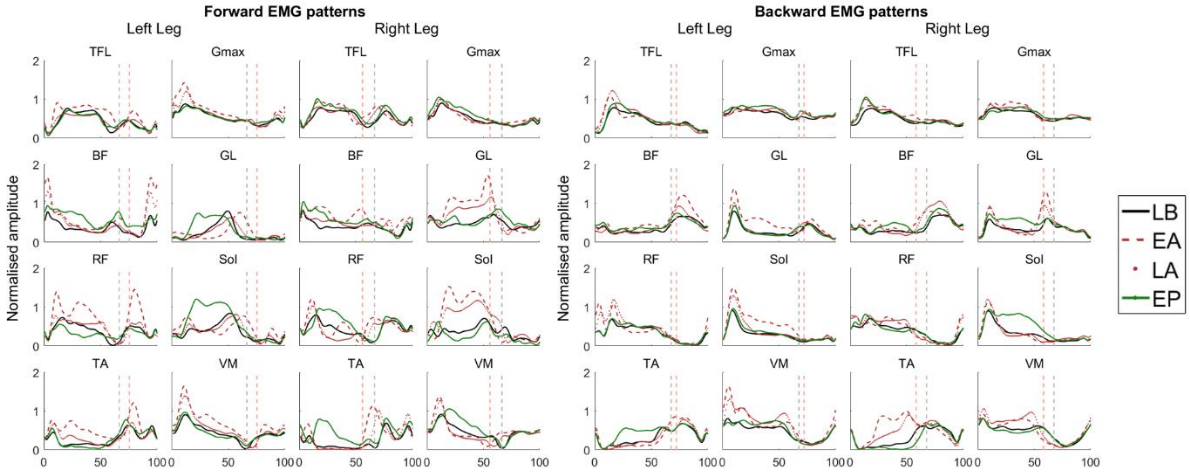

Fig S5. EMG patterns for FW and BW adaptation. The plots present the normalized EMG activations recorded from the 16 EMGs (8 per leg) that were acquired during the FW (left panel) and BW (right panel) experiments. In each single plot, the curves represent the average (across subjects and steps) EMG activations normalized to the maximum value of each muscle at baseline for each subject. In each plot the solid black line represents the EMG activity during late baseline (LB), the dashed red line represents the activity during early adaptation (EA), the dotted red line represents the activity during late adaptation (LA) and the green line represents the activity during early post-adaptation (EP). In each plot, the vertical dashed lines represent, for FW, the instant of toe-off during late baseline (black) and early adaptation (red), while for BW the vertical dashed lines represent the instant of heel-off during late late baseline (black) and early adaptation (red).

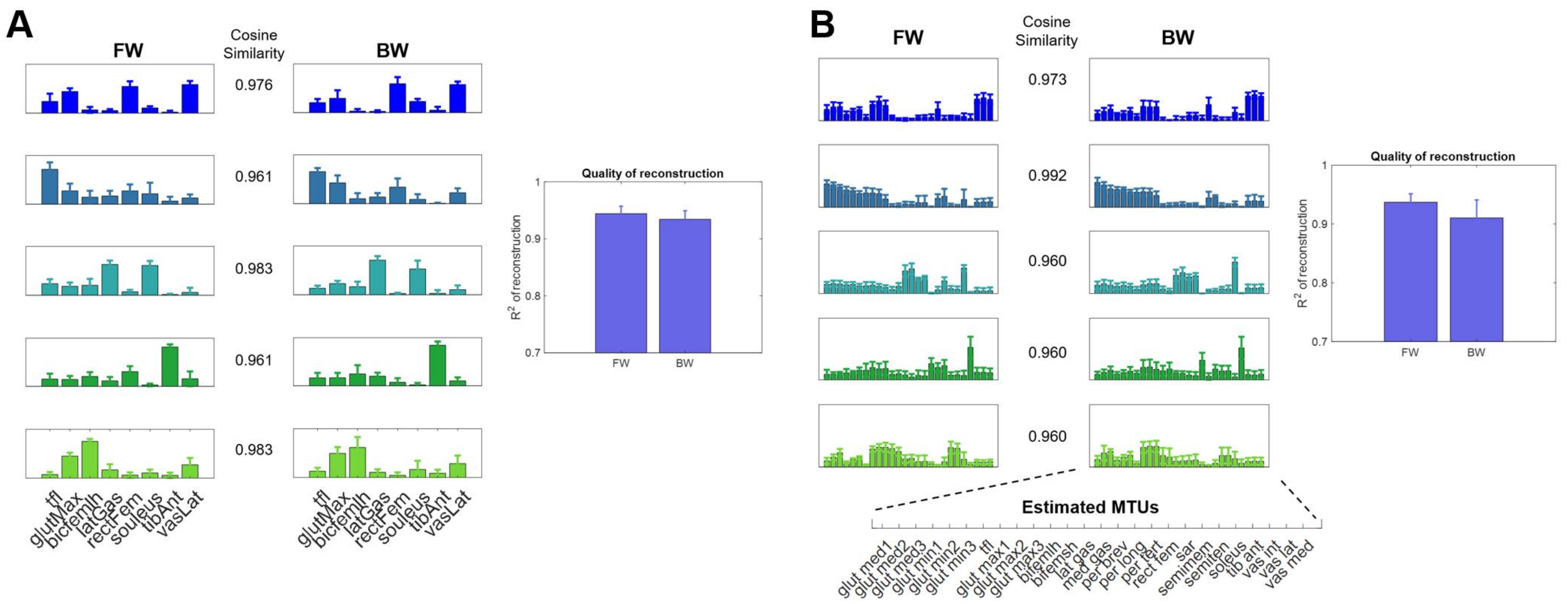

Fig S6. Synergy modules extracted at baseline from the recorded EMGs and the reconstructed MTUs. Panel (A) presents the synergies extracted from the recorded EMGs, while panel (B) presents the synergies extracted from the MTUs. In each panel, the two columns present the synergy modules obtained by applying the standard non-negative matrix factorization algorithm to the baseline data recorded in the FW (left column) and BW (right column) experiments. In each subplot, each bar presents the average (across legs and subjects) of the synergy weights for each module. The value of cosine similarity between each average (across legs and subjects) module for FW and BW is presented. The rightmost bar plot in each panel presents the average and standard deviation (across legs and subjects) quality of reconstruction for the synergies extracted, expressed using the R^2 value calculated between the actual EMG envelopes and those reconstructed from the synergies.

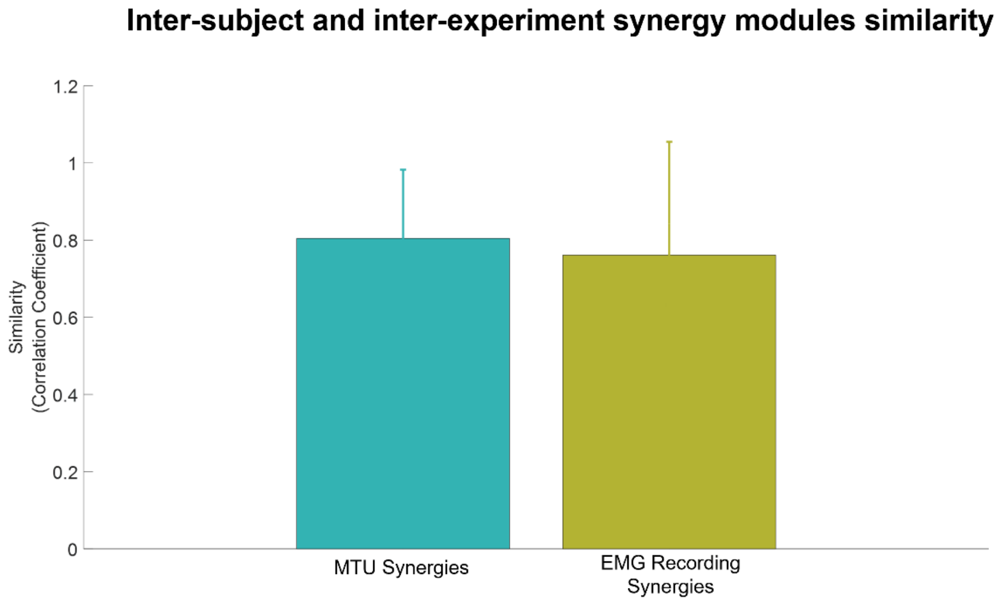

Fig S7. Inter-subject and inter-experiment synergy modules similarity. The two bars represent the average and standard deviation of the similarity (calculated using the cosine similarity) between the synergy modules at baseline for each leg of each subject during one experiment and all the corresponding synergies for each leg of all the other subjects at baseline of both FW and BW. In total, each bar plot represents the average and standard deviation of 2760 comparisons across subjects and conditions. The left bar represents the comparison performed on the synergies extracted from the estimated MTUs, while the right bar represents the same comparison performed for the synergies extracted from the 16 EMG signals.

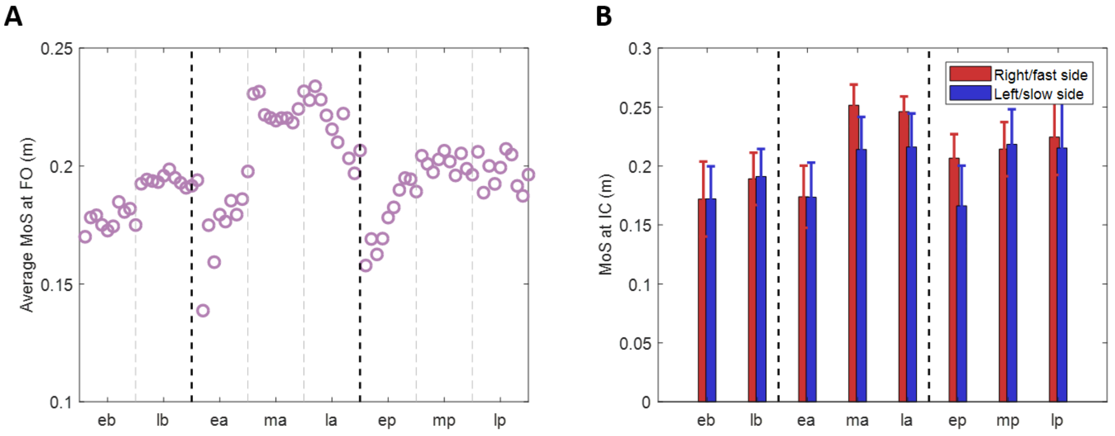

Figure S8. MoS analysis for FW, comparison with previous literature. Panel (A) shows the average, across legs and subjects, MoS calculated at FO, to be compared with [1]. Panel (B) shows the average across the 10 steps of each phase MoS for each leg calculated at IC, to be compared with [2].

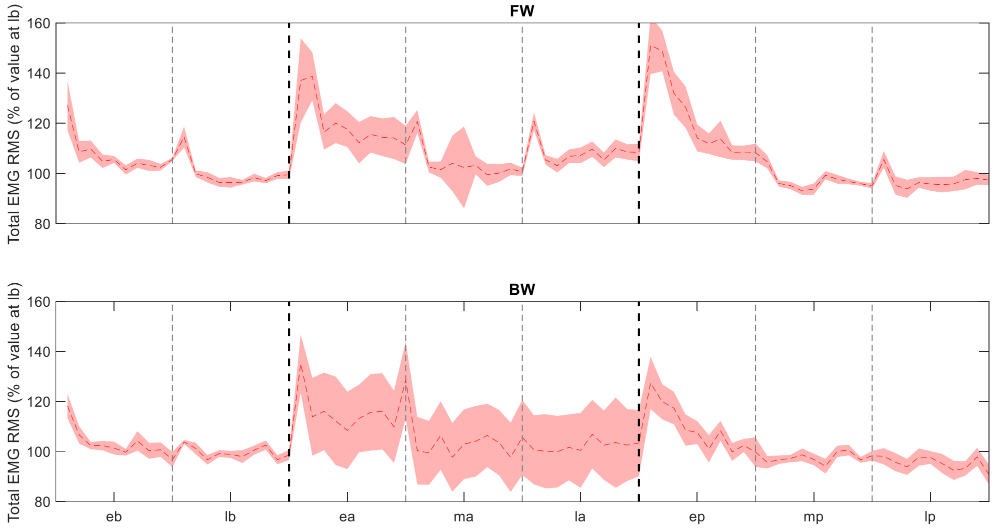

Figure S9. Overall recorded EMG activity. The overall EMG activity was estimated as the sum of the Root Mean Square of each of the 16 recorded EMG channels during each analyzed step. The values are expressed as a percentage of the average total RMS at *lb.* The top plot presents the results for the FW experiment, while the bottom plot presents the results for the BW experiment.

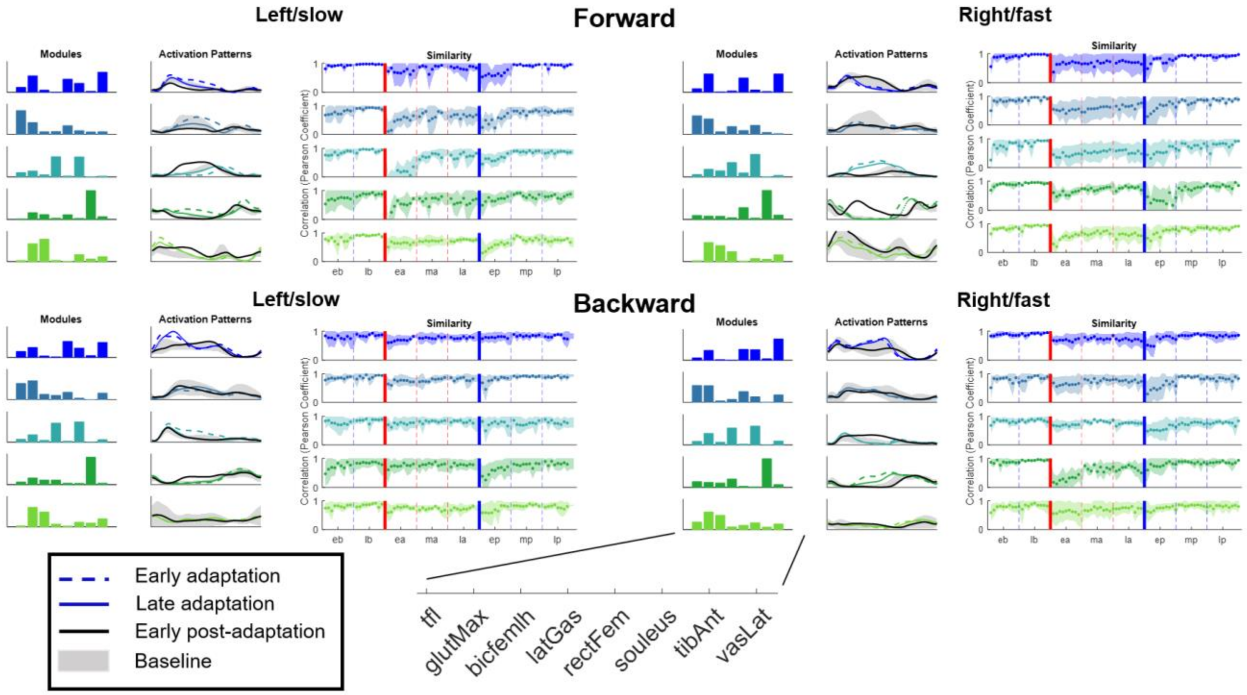

Fig S10. Synergies adaptation calculated from the synergies extracted from the recorded EMGs. The four panels show the adaptation behaviors for each side (left, slow and right, fast) and each experiment (FW top, BW bottom), for the recorded EMG, in the same analysis shown in Figure 2. Each panel shows, from left to right: first plot - the average (across subjects) modules extracted at baseline; second plot - the average (across subjects) activation patterns during specific phases of the experiment (the dashed lines represent the average during all the steps of early adaptation, the solid line the average during late adaptation, the black line the average during early post-adaptation; the gray shaded area represents the average ± standard deviation during baseline); third plot - the average (across subjects) similarity values (calculated using Pearson's correlation coefficient) derived from the average baseline activation pattern of each synergy and the activation pattern of each step in the different phases of the experiment. Shaded areas represent the standard deviation across subjects. The vertical solid lines represent the transition between baseline and adaptation (red) and between adaptation and post-adaptation (blue). The vertical dashed lines represent the transitions between the different sub-phases of each phase.

Table S1. Average (across subjects and steps) ± standard deviation (across subjects) values of MoS and MoS to BoS ratio for late baseline (LB), early and late adaptation (EA, LA) and early and late post adaptation (EP, LP) for both experiments.

		LB		*EA*		*LA*		*EP*		*LP*	
		Left	*Right*	*Left*	*Right*	*Left*	*Right*	*Left*	*Right*	*Left*	*Right*
FW	MoS [cm]	20 ± 1	20 ± 1	11 ± 1	24 ± 2	22 ± 2	21 ± 2	24 ± 4	12 ± 1	20 ± 2	19 ± 2
	Ratio %	52 ± 6	52 ± 7	48 ± 20	42 ± 2	61 ± 5	37±3	52 ± 10	42 ± 16	54 ± 4	52 ± 6
BW	MoS [cm]	14 ± 1	13 ± 3	15 ± 3	17 ± 2	20 ± 2	14 ± 2	18 ± 3	11 ± 2	14 ± 1	13 ± 1
	Ratio [%]	40 ± 2	36 ± 5	48 ± 20	33 ± 4	48 ± 5	30 ± 4	45 ± 5	38 ± 9	38 ± 4	39 ± 5

Table S2. Weight coefficients of the different MTUs onto the recorded muscles.

	Gmax2	TFL	VL	BFl	RF	Soleus	TA	GL
Gmed1	0.2	0.6						
Gmed2	0.4	0.4						
Gmed3	0.6	0.2						
Gmin1	0.2	0.6						
Gmin2	0.4	0.4						
Gmin3	0.6	0.2						
Gmax1	1							
Gmax2	1							
Gmax3	1							
TFL		1						
VL			1					
VM			1					
VI			1					
BFl				1				
BFs				1				
Semimem				1				
Semiten				1				
Sar				0.5	0.5			
RF					1			
Perbrev						0.5		
Perlong						0.5		
PerTert							0.5	
Soleus						1		
TA							1	
GL								1
GM								1
